# Control of Nanoparticle Size of Intrinsically Fluorescent PET (Polyethylene Terephthalate) Particles Produced Through Nanoprecipitation

**DOI:** 10.3390/molecules30020282

**Published:** 2025-01-13

**Authors:** Raffaella Lettieri, Muhammad Mudassir, Fabio Domenici, Andrea Salina, Mariano Venanzi, Cadia D’Ottavi, Elisabetta Di Bartolomeo, Emanuela Gatto

**Affiliations:** Department of Chemical Science and Technologies, University of Rome Tor Vergata, 00133 Rome, Italy; muhammad.mudassir@students.uniroma2.eu (M.M.); fabio.domenici@uniroma2.it (F.D.); andrea.salina@alumni.uniroma2.eu (A.S.); venanzi@uniroma2.it (M.V.); d.ottavi@scienze.uniroma2.it (C.D.); dibartolomeo@uniroma2.it (E.D.B.)

**Keywords:** polyethylene terephthalate nanoparticles, fluorescence spectroscopy, nanoplastic, dynamic light scattering

## Abstract

Plastics are widely produced due to their stability and ease of manufacturing, but many of them quickly become a waste, breaking down into microplastics and nanoplastics. While methods for the identification and characterization of plastic particles are well consolidated, the small size of nanoplastics presents challenges for their detection and analysis. Furthermore, due to the difficulty of identifying nanoplastics, analytical studies concerning their effect on cells and a comprehensive spectroscopic characterization are still lacking. In this paper, we overcome this obstacle by synthesizing and characterizing, for the first time, PET nanoparticles with specific, stable dimensions through a top-down approach. Using hexafluoroisopropanol-chloroform as a solvent, we prepared PET solutions at various concentrations and analyzed their spectral properties over time. Our results show that PET aggregates into nanoparticles, the quantity of which increases with concentration. These findings provide crucial insights for the detection of nanoplastics in environmental samples through fluorescence measurements and can potentially be used to produce stable PET nanoparticles to evaluate their cytotoxicity.

## 1. Introduction

Global plastics production reached 400.3 million tons in 2022 [1], of which approximately 4.8 to 12.7 million tons are poured into the oceans every year [2]. It has been estimated that only 9% of all plastics produced gets recycled, while about 60% ends up in landfills or is dispersed in the environment [3]. The reckless use of plastics in our daily life has made this material the main pollutant of the world’s seas and oceans [4,5,6,7].

The problem is that plastics are very stable materials and do not biodegrade in the environment. Plastics degradation takes place through abiotic mechanisms, such as photo, thermal, and chemical degradation and mechanical abrasion [8,9]. But the effect of degradation over time is that it fragments into small particles: microplastics (MPs) and nanoplastics (NPs) [10,11,12,13,14]. For this reason, a huge number of MPs and NPs are found in sediments, in coasts, and also in water. MPs are solid plastic particles with dimensions less than 5 mm [9], composed of mixtures of polymers and functional additives. They may also contain residual impurities. MPs can be formed not only when larger pieces of plastic, like car tires or synthetic textiles, wear and tear, but they are also deliberately manufactured and added to products for specific purposes, such as exfoliating beads in facial or body scrubs [15].

MPs can have different shapes such as spheres, fibers, and fragments. Moreover, MPs can be further degraded into NPs, which are particles of sizes from 1 to 1000 nm [16]. MPs and NPs found in the sea can be ingested by marine animals [17,18]. The plastic then accumulates in the organisms and can end up in humans through the food chain. Otherwise, they can enter human bodies through the skin, digestive, genital, and respiratory mucosae [19,20]. Several studies reported the toxicity of MPs and NPs for human health [21,22].

Because MPs and NPs are very complex mixtures of materials and the established analytical methods are not sufficiently adapted and validated, there is currently a lack of reliable methodologies for the validation of MPs and NPs dispersed in the environment. In particular, various methods have been developed in the literature for the study and characterization of different microplastics [23,24,25,26,27,28,29,30,31,32,33], but the small size of NPs limits the use of standard characterization techniques (IR and Raman), especially when there are several different plastic polymers in the same sample [34,35].

Furthermore, micrometric particles found in the environment cannot be used to study the cytotoxic effect on living cells, for two reasons. The first is that the dimensions of human cells are micrometric, so using microparticles can be very difficult to distinguish if possible effects on cytotoxicity are due to their chemical or physical properties [36]. Second, plastic particles are going to change their chemical composition over time due to the ageing of the sample, and a systematic study of the effect of the different polymers on the cells is difficult to perform.

To assess the health impact of different types of polymers, it is necessary to obtain pure polymer NPs with well-known and reproducible characteristics (for example, shape, dimensions, and stability over time). Additionally, a technique is needed to determine the quantity and type of NPs in environmental samples.

Within plastic polymers, PET represents 6.2% of the plastic demand in the world. PET is a semi-crystalline polyaromatic polymer with the empirical formula (C_10_H_8_O_4_)_n_. Interestingly, solutions of polyethylene terephthalate particles show very characteristic absorption and fluorescence emission spectra, but the literature on this topic is very limited. We believe instead that absorption and fluorescence detection methods are very interesting and promising for the recognition and characterization of PET in environmental and biological samples, due to its intrinsic fluorescence. In this work, we have developed a simple method to produce PET (Polyethylene terephthalate) nanoparticles, starting from commercial-grade PET granules, by using hexafluoro isopropanol (HFIP) and chloroform 1:1 (*v*/*v*). In general, HFIP is the preferred solvent for PET solubilization, because it provides fast solubilization at room temperature [37] and is transparent to UV light [38]. PET NPs were produced by Johnson et al. with a precipitation method in which ultrapure water was added to a solution of PET in HFIP to study their effects in biological systems [39]. However, since HFIP is expensive and irritating to the skin and eyes [40], it is preferable to reduce the amount of HFIP using a mixture of solvents. In this work, in order to induce nanoprecipitation, the quality of the solvent in which the polymer is dissolved was reduced by altering the solubility conditions using a second solvent with a lower dielectric constant. Within the solvents, we have selected chloroform, which reduces the solubility of PET in HFIP, facilitating the formation of PET nanoparticles.

We have prepared PET NPs starting from the commercial resin, and we have tested their stability over time and characterized them by spectroscopic (UV-visible absorption, fluorescence, and dynamic light scattering) and microscopic techniques (scanning electron microscopy and optical microscopy), correlating their dimensions to their spectral properties.

The synthetic method of this work is fast and inexpensive and can potentially be applied to the study of nanoparticle–cell interactions, thus more conveniently assessing the cytotoxicity of PET polymers. These findings provide insight for the detection of nanoplastics in environmental samples through fluorescence and phosphorescence measurements and for the production of stable PET nanoparticles to evaluate their cytotoxicity.

## 2. Materials and Methods

### 2.1. Materials

Spectro-grade solvents (Carlo Erba, Cornaredo, Italy) were used exclusively. Chemicals were all of reagent-grade quality and were used without further purification.

### 2.2. PET Nanoparticles Preparation

**Figure 1:**
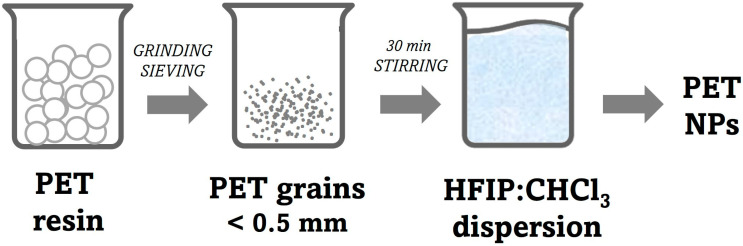
Schematic description of the PET nanoparticles production.

Millimetric granules of commercial-grade PET resin (Relpet, Jamnagar, India) were used to produce nanometric particles. The granules were ground for 30 min in a laboratory grinder. The obtained particles were then further processed by separating the finer part with a sieve (0.5 mm mesh size). The fine powder obtained was dissolved at room temperature in the organic solvents mixture HFIP:CHCl_3_ 1:1 (*v*/*v*) after 30 min of stirring (Figure 1). Different concentrations were prepared, ranging from 0.8 to 1.8 mg/mL. For each characterization technique, the samples were prepared in triplicate.

### 2.3. Spectroscopic Measurements

For spectroscopic analysis, different PET concentrations were prepared, i.e., 0.8, 1.0, 1.4, 1.8 mg/mL in HFIP:CHCl_3_ 1:1 (*v*/*v*). UV-visible absorption measurements were carried out at room temperature using a Varian Cary 100 Scan spectrophotometer (Middelburg, The Netherlands). The absorption spectra were recorded using a 1 mm path length quartz cuvette. Steady-state fluorescence measurements were carried out using a Fluoromax-4 (HORIBA, Jobin Yvon, Milano, Italy) spectrofluorometer, using a 0.5 × 0.5 cm quartz cuvette at 25 ± 0.1 °C, controlled by a thermostatted cuvette holder. Fluorescence emission spectra were measured at the excitation wavelengths of 275, 290, and 340 nm. All spectra were recorded using 2 nm bandwidths for both the excitation and emission monochromators, an integration time of 1 s, with an increment of 1 nm. The spectra were blank-subtracted.

### 2.4. PET Nanoparticles Characterization

Fluorescence microscopy measurements were obtained using an Axio Scope microscope ZEN 3.8, (Carl Zeiss MicroImaging Gmbh, Göttingen, Germany) equipped with a CCD AxioCam ICm1 camera and a mercury lamp HBO 50. A volume of 0.1 mL of the 1.8 mg/mL PET solution was dropped onto a glass microscope slide and the analyses were performed after 1 h of waiting for the complete evaporation of the solvent at room temperature. The sample was excited with UV light, selecting DAPI filter, and the emission was obtained in the blue range (the excitation maximum for DAPI is at 358 nm and the emission maximum is at 461 nm).

The morphology of PET nanoparticles was investigated using the Field Emission Scanning Electron Microscope (FE-SEM). A 50 μL volume of the dispersion, prepared by dissolving 1 mg/mL PET in HFIP:CHCl_3_ 1:1 (*v*/*v*), was placed on a coverslip glass and dried in an oven at 50°C for 30 min. After drying, the coverslip was sticked on a carbon tape on the top of a SEM disk and the sample was sputter-coated with gold for 1 min. The sample was analyzed using a FE-SEM Zeiss Leo SUPRA^TM^ 35 (Carl Zeiss SMT, Oberkochen, Germany). Image J software version 1.54g was used to count and measure the particles.

DLS measurements were performed with a Zetasizer Nano ZS (Malvern, Alfatest, Roma, Italy) operating at an angle of detection of 173°. Glass cuvettes were used for experiments. The measurements were made at a controlled temperature of 25 °C. For each sample, six measurements were averaged.

## 3. Results and Discussion

### 3.1. Characterization of the System in Solution

**Figure 2:**
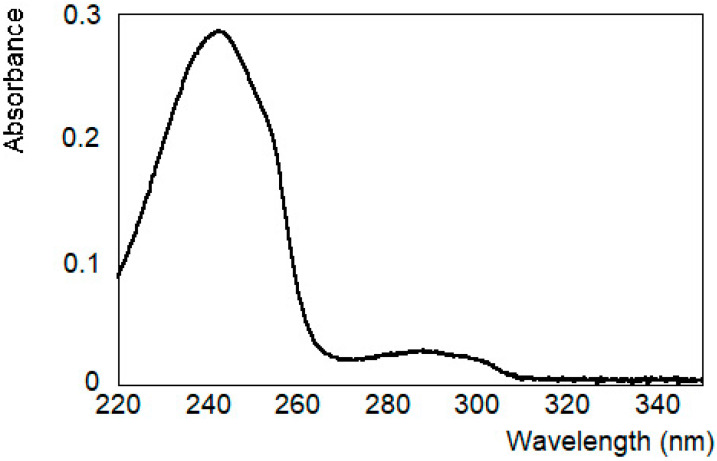
Spectroscopic features: absorption spectrum of PET solution in HFIP: CHCl_3_.

**Figure 3:**
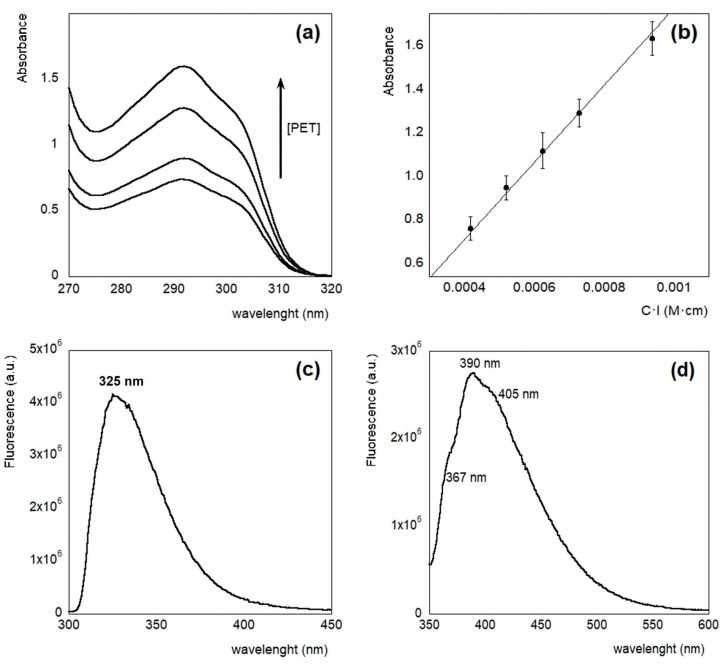
Spectroscopic features of PET solutions in HFIP: CHCl_3_: (**a**) absorption spectra; (**b**) Lambert-Beer plot; (**c**) emission spectrum with λ_ex_ = 290 nm of PET 0.8 mg/mL in HFIP:CHCl_3_; (**d**) emission spectrum of PET 6 mg/mL in HFIP:CHCl_3_, λ_ex_ = 340 nm.

The spectroscopic features of the PET dispersions in HFIP:CHCl_3_ 1:1 (*v*/*v*) are reported in Figure 2. The signal is similar to the one reported for PET in HFIP solution [41,42]. It is characterized by the ^1^A→^1^L_b_ transition, with a maximum absorption at 290 nm and a shoulder at 304 nm, and another maximum at 244 nm, corresponding to the ^1^A→^1^L_a_ transition. In the literature, an absorption band at 197 nm is also reported, assigned to the ^1^A→^1^B transition [43,44]. We decided to follow the absorption intensity of PET dispersions in HFIP:CHCl_3_ 1:1 (*v*/*v*) at different concentration values, in a wavelength range in which the linearity of the signal is observed (270–320 nm). Figure 3a shows the absorption spectra in the near-UV region, at different concentrations ranging from 0.8 to 1.8 mg/mL (concentration of PET monomer: 4.2 and 9.4 mM). Compared to the PET film, a red shift of 10 and 14 nm of the two transitions was observed, respectively [45]. From the Lambert–Beer plot (Figure 3b), we have determined a molar extinction coefficient value of ε (290 nm) = (1775 ± 13) M^−1^cm^−1^. This value is similar to the one reported for Dimethyl Terephthalate (DMT) by Wagener et al. in HFIP solution [46].

The fluorescence spectrum of PET dispersion in HFIP:CHCl_3_ solution, in the near-UV region, was obtained by exciting the sample at 290 nm. As reported in Figure 3c, it shows an emission maximum at a wavelength of 325 nm. These features are very similar to the ones reported by Frank et al. [47] for the monomeric form of the PET polymer, namely, the dimethyl terephthalate (DMT) crystals, as well as for PET solutions in trifluoroacetic acid (TFAA) and dichloroacetic acid (DCAA), which also show an emission maximum at 325 nm. The authors attributed this signal to the monomer unit of PET. To characterize the system by varying both the concentration and the age of the solution, the spectra were recorded for four different HFIP:CHCl_3_ 1:1 (*v*/*v*) PET solutions in a concentration range between 0.8 and 1.8 mg/mL, and day 1, 3, 5, and 8 of the preparation.

**Figure 4:**
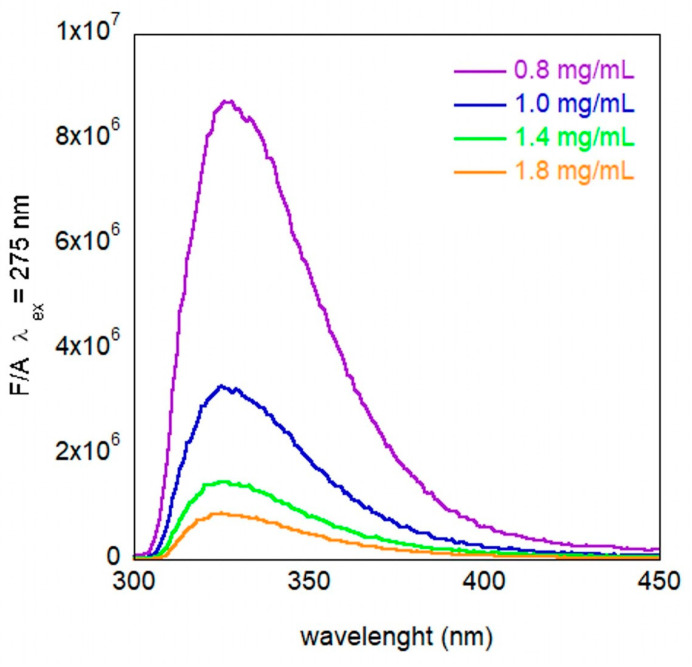
Emission intensity divided by the absorption at the excitation wavelength value of PET in HFIP:Chloroform 1:1 (*v*/*v*), with λ_ex_ = 275 nm in fresh samples at different concentrations.

**Figure 5:**
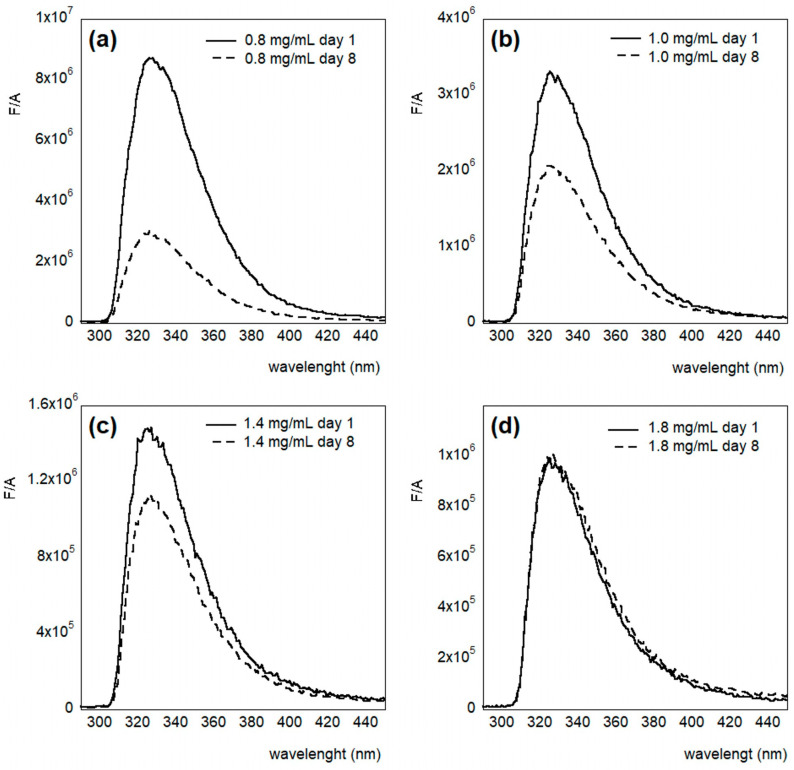
Emission spectra evolution over time (on day 1 and on day 8) of PET in HFIP:CHCl_3_ 1:1 (*v*/*v*), λ_ex_ = 275 nm, at different concentrations: (**a**) 0.8 mg/mL solution; (**b**) 1.0 mg/mL solution; (**c**) 1.4 mg/mL solution; (**d**) 1.8 mg/mL solution. The spectra recorded on day 1 and on day 8 are indicated by full and dashed line, respectively.

Figure 4 shows the obtained fluorescence spectra obtained from the four different concentrations, divided by the absorption value at the excitation wavelength. We observed that this signal decreases by increasing the concentration. Furthermore, Figure 5a–d show the fluorescence spectra of the four different concentrations in time. It should be noted that the emission intensity is higher in fresh samples, compared to the older ones (Figure 5a–c), while in the more concentrated solutions, the intensity is almost the same (Figure 5d). This result suggests aggregation of the PET by improving the concentration and over time. This process is in equilibrium with the more concentrated solution, where no kinetics can be detected.

Frank et al. [48] also reported a phosphorescence signal upon exciting the dimethyl terephthalate (DMT) crystals and PET solutions in TFAA and DCAA at 340 nm. Interestingly, they found that PET in DCAA, in which it is not soluble, shows an intense spectrum with an emission maximum at 390 nm, and with two shoulders at 367 nm and 405 nm, which is very similar to that of the PET film. The PET in TFAA, on the other hand, in which it is soluble, shows a less intense spectrum, with an emission maximum at 460 nm. To check the phosphorescence of our samples, we also performed spectra in this region, exciting the sample at 340 nm. The emission spectrum of a high concentration solution (6 mg/mL) of PET in HFIP:CHCl_3_ 1:1 (*v*/*v*) is reported in Figure 3d. The spectrum shows an emission maximum at 390 nm, with two shoulders at 367 nm and 405 nm. These signals are very similar to the spectral features of PET solution in DCAA, in which the PET is not soluble, or those of the unoriented PET polymer film [41,48]. Interestingly, in these experiments, the authors attributed the emission maximum at 368 nm to a fluorescing structure called “trap”. The trap can be excited by energy migration to this structure of the monomer emission or by its direct absorption at 340 nm. This trap has been interpreted as a ground state dimer [40] or as an aggregate, which, in general, in PET films, is located in the amorphous phase of the semi-crystalline polymer [47,49].

To understand whether the previously described fluorescence decrease in emission at 325 nm could be attributed to some aggregation phenomenon, we measured the emission spectra of four different HFIP:CHCl_3_ 1:1 (*v*/*v*) PET solutions also by exciting the samples at 340 nm.

**Figure 6:**
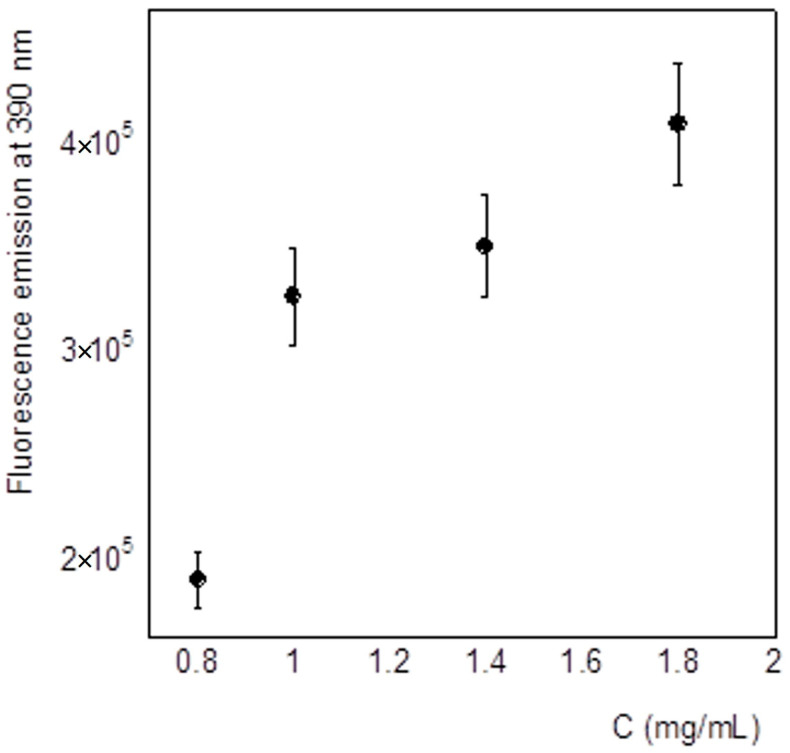
Concentration dependence of the fluorescence emission intensity at λ_em_ = 390 nm of PET in HFIP:CHCl_3_, λ_ex_ = 340 nm.

Interestingly, we found that the emission at 390 nm of the freshly prepared solutions is not proportional to the concentration (Figure 6). In particular, the graph suggests that by improving the concentration, the quantity of the aggregate improves, but reaches a plateau value.

**Figure 7:**
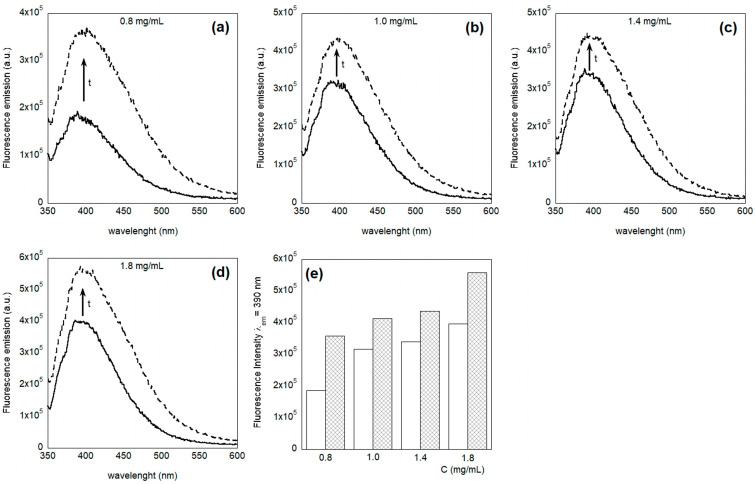
Fluorescence spectra of PET in HFIP:CHCl_3_ 1:1, v:v at different concentrations: (**a**) 0.8 mg/mL (**b**) 1.0 mg/mL, (**c**) 1.4 mg/mL, (**d**) 1.8 mg/mL, λ_ex_ = 340 nm; the spectra were recorded in fresh solutions (solid line) and aged solutions after 8 days (dotted line), as is also summarized in the histogram in figure (**e**) that reports the fluorescence values at λ_em_ = 390 nm, comparing fresh solutions (white columns) to aged solutions (checkered columns).

The same measurements performed following the samples over time showed that the fluorescence intensity at 390 nm increased for all the samples, i.e., the intensity of the emission obtained after 8 days was higher than the one obtained with fresh samples, independently of the concentration of the solution (Figure 7). This result suggests that the quantity of the aggregates increased during the time in all of the solutions analyzed. This observation, together with the observation of a decrease of the monomer fluorescence, suggests that the PET, under the experimental conditions used, is going to aggregate.

**Figure 8:**
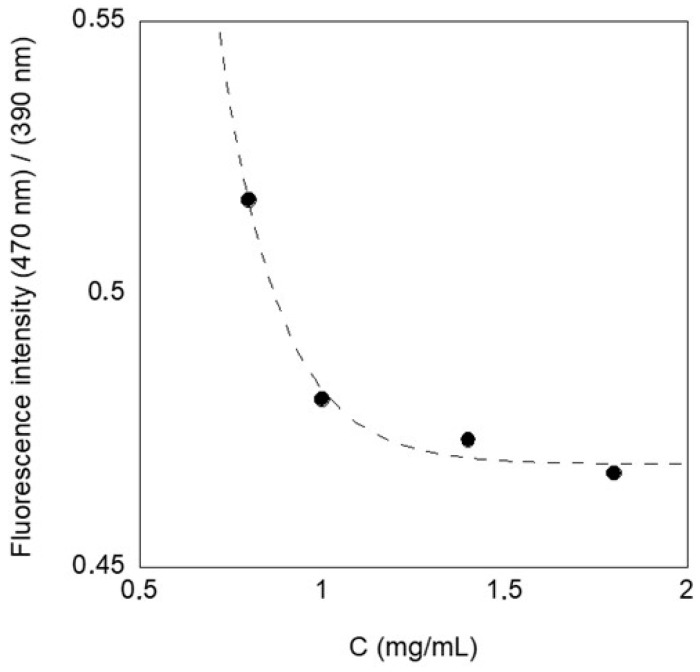
Ratio I (470 nm)/(390 nm) of the emission spectra of PET at different concentrations, λ_ex_ = 340 nm, considering the values obtained on day 8.

To demonstrate this, we reported the ratio between the fluorescence signal at 470 nm, which is attributed to the PET monomer, and that at 390 nm, attributed to the aggregate (Figure 8) in function of the concentration of the solution, at the equilibrium time; that is, after 8 days from the preparation. We observed a decrease of this value, in function of the concentration, suggesting that by increasing the concentration, the amount of aggregates increases in solution.

This result suggests that aggregates are formed in solution, reducing the fluorescence of the monomer and improving the signal of the aggregated species, and that this aggregation process has a slow kinetic in less-concentrated solution.

The fluorescence signals that we observed are very similar to the emission spectrum of PET films reported in the literature, indicating that this approach can be used to identify PET particles in environmental samples. In particular, Padhye and Tamhane have reported the spectrum of a PET film obtained at 77 K [45]. Under the applied experimental conditions, the authors reported three distinct luminescence bands peaked at 304, 326, and 365 nm [44], and demonstrated that the emitter is the terephthalate unit. Furthermore, this study suggested that the three different bands arise from different environments of the emitting center and that the origin of the band at 365 nm was the cis form of the PET; that is, the amorphous phase, while the 326 nm band originated from the trans form of the PET related to the crystalline form.

Our results are in accordance with the spectrum obtained in this region and suggest that the PET is in its crystalline state, since there is no high signal at 368 nm. Furthermore, the authors also reported three different peaks at 426, 453, and 476 nm, corresponding to the phosphorescence signal. We also observed a phosphorescence signal, indicating that PET dissolved in a solution of HFIP:CHCl_3_ is in aggregate form. Our fluorescence results suggest that the PET grains are going to be well dissolved in HFIP:CHCl_3_ solutions, but that the solute is going to aggregate over time, giving rise to increased fluorescence signals in the wavelength range between 360 and 410 nm [48].

Further characterization of the PET NPs was achieved using dynamic light scattering (DLS). For similar systems, obtained by Johnson et al. by dissolving PET in HFIP with the addition of water, a hydrodynamic diameter of 158 ± 2 nm was obtained [39]. NanoPET particles ranging from 50 to 300 nm were detected by Rodriguez et al. [50].

**Figure 9:**
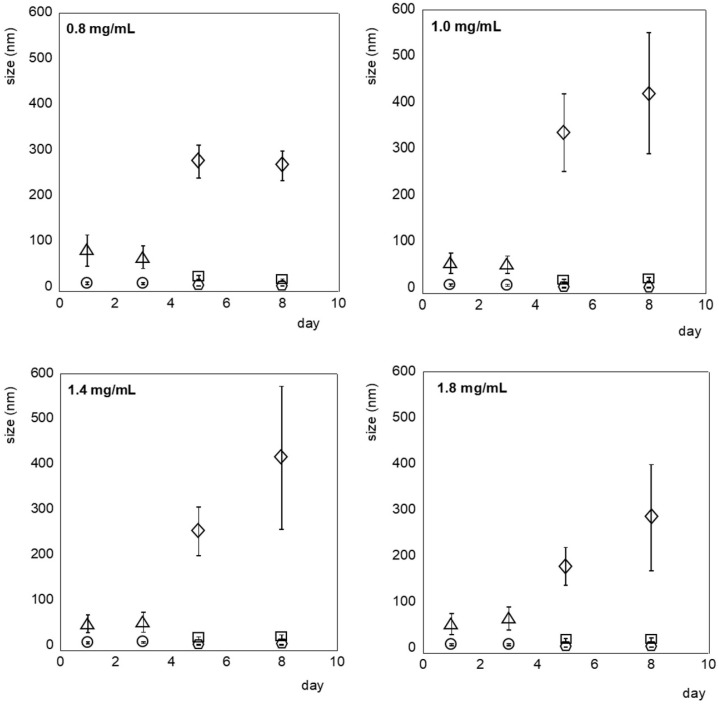
DLS analyses of PET aggregates formed in HFIP:CHCl_3_ at different concentrations (0.8, 1.0, 1. 4, 1.8 mg mL^−1^). The size is reported as a function of time. Circles are referred to agglomerates of 7–8 nm size; triangles are referred to agglomerates of 15–80 nm size; squares are referred to smaller size species of 1–2 nm; rhombus are referred to agglomerates of 250–550 nm size.

**Figure 10:**
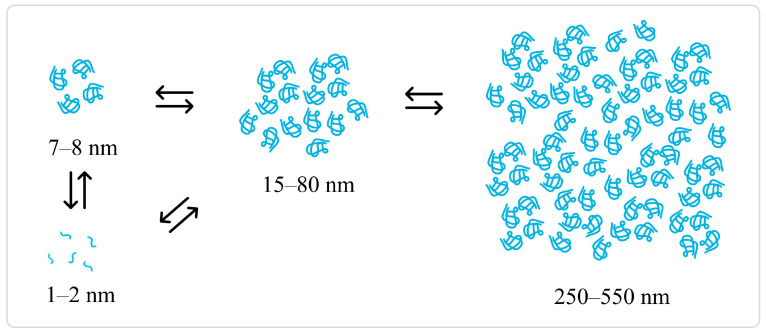
Aggregation model for PET nanoparticles in HFIP/CHCl_3_. From the first day of sample preparation until the third day, the data suggested that two groups of agglomerates formed by PET oligomers (7–8 nm, and 15–80 nm) are present, regardless of the initial concentration between 0.8 and 1.8 mg/mL. After 5 days, a differentiation of the aggregates occurs: a smaller size (1–2 nm), probably formed by isolated unimers, is observed, and an intermediate size in the range 15–80 nm, together with larger agglomerates (250–550 nm), are detected.

DLS investigations of PET dispersed in HFIP:CHCl_3_ 1:1, (*v*/*v*) (0.8, 1.0, 1.4, 1.8 mg/mL) were performed on fresh solutions and repeated 3, 5, and 8 days after the sample preparation, as shown in Figure 9, in which the size of the particles is reported as a function of time for each concentration. Complete analysis of the size distribution by intensity, by volume, and raw correlation data are reported in Supplementary Materials, Figures S6.1–S6.16). The more diluted solution (0.8 mg/mL) showed two distributions characterized by the average hydrodynamic diameter of PET nanoparticles of 8 ± 3 nm and 79 ± 34 nm, respectively. Considering that the size of the terephthalic acid unit, calculated using the software Chimera 1.17.3, is 8 Å, it is reasonable to assign the smaller particle size to oligomeric clusters of the unimeric folded form of PET, and the other size to agglomerates formed by oligomers. After 3 days, no substantial modification could be observed. Moving from 3 to 5 days, it was observed that three main size distributions were present: 1.9 ± 0.4, 21 ± 4, and 275 ± 36 nm. The measurements performed after 8 days confirmed the presence and stability in time of these three main aggregate types. DLS analyses performed on more concentrated samples (1.0, 1.4, and 1.8 mg/mL) showed similar behavior on a shorter period: two “families” of aggregates with an average hydrodynamic diameter size of around 7 and 50 nm were observed from the first day of preparation until the third day of measurement. The size of these species did not change with the concentration of the solution. These results suggest that the aggregation is of “close type”, indicating that even if the number of aggregates change, their size does not (Figure 10). After 5 days, the formation of both smaller and larger aggregates can be detected. However, unlike the 0.8 mg/mL solution, the larger aggregates tend to grow over time, and the standard deviation associated with the bigger size increases, suggesting that the nanoparticles are no longer stable.

These results are very promising because, through a very simple, rapid, and economical method, it was possible to obtain polymeric particles of defined dimensions that are stable over a useful time interval. These, being composed of a pure polymer, can therefore be easily used as a reference for further studies of the toxicity of nanoplastics toward cells.

### 3.2. Characterization of PET NPs on Surface

**Figure 11:**
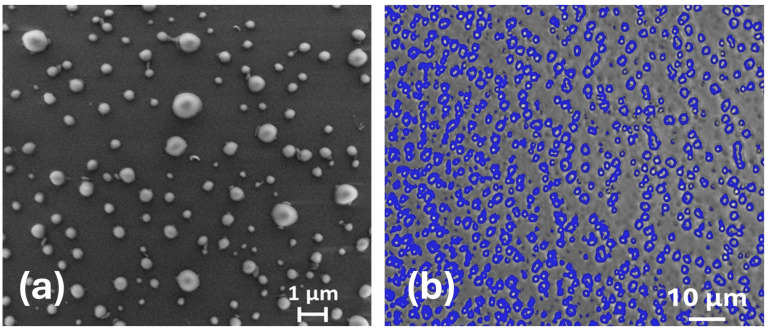
(**a**) SEM micrograph showing the regular spherical shape of PET nanoparticles; (**b**) fluorescence microscopy imaging of PET nanoparticles.

Although DLS is a powerful technique for characterizing a dispersion of nanoparticles, a determination of the shape of the aggregates is not achievable from this method alone [51]. To investigate the shape of the obtained PET nanoparticles, we also performed surface characterization using scanning electron microscopy (SEM). SEM analysis revealed spherical particles with diameters ranging from 10 to 200 nm, observed starting from the 1.8 mg/mL PET solution, confirming the production of PET nanoparticles (Figure 11a). The presence of a layer of PET that forms like a film after solvent evaporation confirms the good solvent properties of the HFIP:CHCl_3_. Interestingly, nanoparticles can be detected also three months after the solution preparation (Figure S9). This suggests that the method we developed is a good procedure for preparing stable PET NPs. The use of Image J software made it possible to collect statistical data and compile a histogram showing the number of particles and their size distribution (Supplementary Materials, Figure S8). A Gaussian fit gave a maximum distribution at 70 nm, with a full width at half maximum of 80 nm, obtained by measuring the diameter of 804 particles in total. This result is similar to what is obtained in the literature for PET NPs in HFIP solution, demonstrating that HFIP:Chloroform is also a very good medium to obtain PET NPs. Comparing these results with the size of the nanoparticles obtained in solution by DLS analyses, the size seems to be slightly lower in SEM. The differences between the hydrodynamic diameters and the diameters calculated from SEM and fluorescence microscopy images (i.e., dried samples) are expected. These differences could result from the indirect measurements associated with DLS, which rely on fluctuations in the particle scattering intensity in solution. Additionally, the hydrodynamic size includes any solvent molecules attached to the surface of the nanoparticles.

Fluorescence microscopy measurements confirmed the formation of a PET film after solvent evaporation, together with spherical particles, suggesting that the solvent mixture HFIP:CHCl_3_ 1:1 (*v*/*v*) has solvent properties good enough to solubilize the PET polymer and, at the same time, also promote the formation of PET NPs. In particular, the images reported in Figure 11b showed a homogeneous coverage of the surface due to the PET layer and particles that range from a few micrometers to lower diameters, not imaged by fluorescence microscopy. Both the layer and the particles showed fluorescence when excited with the UV channel, showing blue light emission, in accordance with spectroscopical analysis in solution. This result is very significant, since it confirms the intrinsic fluorescence of the PET particles produced, which can be used for toxicology applications.

Using spectroscopic measurements performed by changing the concentration of HFIP:Chloroform 1:1 (*v*/*v*) PET solutions and following them over time, some conclusions about the aggregation processes occurring in this system can be drawn. The fluorescence spectra reported in Figure 3, d show that PET emission in a solution of HFIP:Chloroform 1:1 is similar to that of PET in DCAA solution or PET film, in which there is a high degree of polymer–polymer interaction. This indicates that, in the solvent mixture used, PET is partially solubilized and, at the same time, the presence of CHCl_3_ promotes the formation of some aggregated species. SEM measurements, fluorescence microscopy, and DLS measurements gave us an indication of the size of the aggregates. In particular, DLS measurements showed the presence of two distributions sizes at the initial stage of preparation: the first one showing an average hydrodynamic diameter of PET nanoparticles of 7–8 nm, and the other one between 15 and 80 nm. The smaller size suggested the presence of small aggregates of PET, while the second of a bigger agglomerate formed by PET oligomers. Over time, the fraction of the larger particles increased, leading to an increase in fluorescence. On the fifth day, the presence of unimeric species took place, giving rise to a decrease of the fluorescence intensity at 390 nm, due to less polymer–polymer interactions. Meanwhile, the 7–8 nm size particles aggregated to bigger size particles of 16–20 nm, while the 80 nm particles grew to 250–550 nm particles. The sizes of these species did not change with varying the concentration of the solution, suggesting a close-type aggregation, in which, even if the number of aggregates changed with the concentration, their size did not. This result is fundamental in the development of PET NPs with defined size that can be used for cell interaction studies and fluorescence spectroscopic analysis. Moreover, the developed method allows for a very fast and inexpensive synthesis of PET nanoparticles displaying stable size and peculiar spectroscopic features, which are suitable for designing a sensor for the detection and analysis of PET nanosized plastics as pollutants in the environment.

## 4. Conclusions

In this work, we set up a protocol for the realization of PET nanoparticles in a mixture of HFIP:Chloroform 1:1 (*v*/*v*). The particles were characterized by UV-visible, fluorescence, and dynamic light scattering spectroscopy and by fluorescence and scanning electron microscopy techniques. Our results demonstrate that, under the experimental conditions used, we were able to generate PET NPs, with nanometric size and spherical shape. The particles produced remain stable for several months. Furthermore, our results show that the PET NPs give rise to a close-type aggregation, in which, by increasing the concentration of PET, the number of particles increases but their size does not.

The applied top-down approach is rapid and inexpensive, and it may be potentially used for the study of nanoparticles–cell interaction to evaluate the cytotoxicity of the PET polymer. Furthermore, this study paves the way for identifying plastic polymers of nanometric dimensions in environmental samples through fluorescent spectroscopy.

## Data Availability

Data are contained within the article and Supplementary Materials.

## Supplementary Materials

The following supporting information can be downloaded at: https://www.mdpi.com/article/10.3390/molecules30020282/s1, UV-vis absorption and fluorescence spectra of PET in HFIP:CHCl_3_ 1:1 (*v*/*v*) at different concentrations, followed over time; absorption and emission deconvolution data; additional details on DLS measurements: size distribution by intensity, by volume, and the raw correlation data; statistical data on the acquired SEM micrographs (docx).
