# Supplementary material for: Control of Nanoparticle Size of Intrinsically Fluorescent PET (Polyethylene Terephthalate) Particles Produced Through Nanoprecipitation
Source: Molecules. 2025 Jan 13;30(2):282. doi: 10.3390/molecules30020282 (PMC11767366; doi:10.3390/molecules30020282)
Supplement: Supplementary file 1 [file molecules-30-00282-s001.zip › molecules-3315682-supplementary.pdf]

Supporting Information of the paper: *Control of nanoparticle size of intrinsically fluorescent PET (Polyethylene terephthalate) particles produced through nanoprecipitation*

Raffaella Lettieri *, Muhammad Mudassir, Fabio Domenici, Andrea Salina, Mariano Venanzi, Cadia D'Ottavi, Elisabetta Di Bartolomeo and Emanuela Gatto *

Department of Chemical Science and Technologies, University of Rome Tor Vergata, 00133 Rome, Italy; muhammad.mudassir@students.uniroma2.eu (M.M.); fabio.domenici@uniroma2.it (F.D.); andrea.salina@alumni.uniroma2.eu (A.S.); venanzi@uniroma2.it (M.V.); d.ottavi@scienze.uniroma2.it (C.D.); dibartolomeo@uniroma2.it (E.D.B.)

* Correspondence: raffaella.lettieri@uniroma2.it (R.L.); emanuela.gatto@uniroma2.it (E.G.); Tel.: +39-06-7259-4469 (R.L. & E.G.)

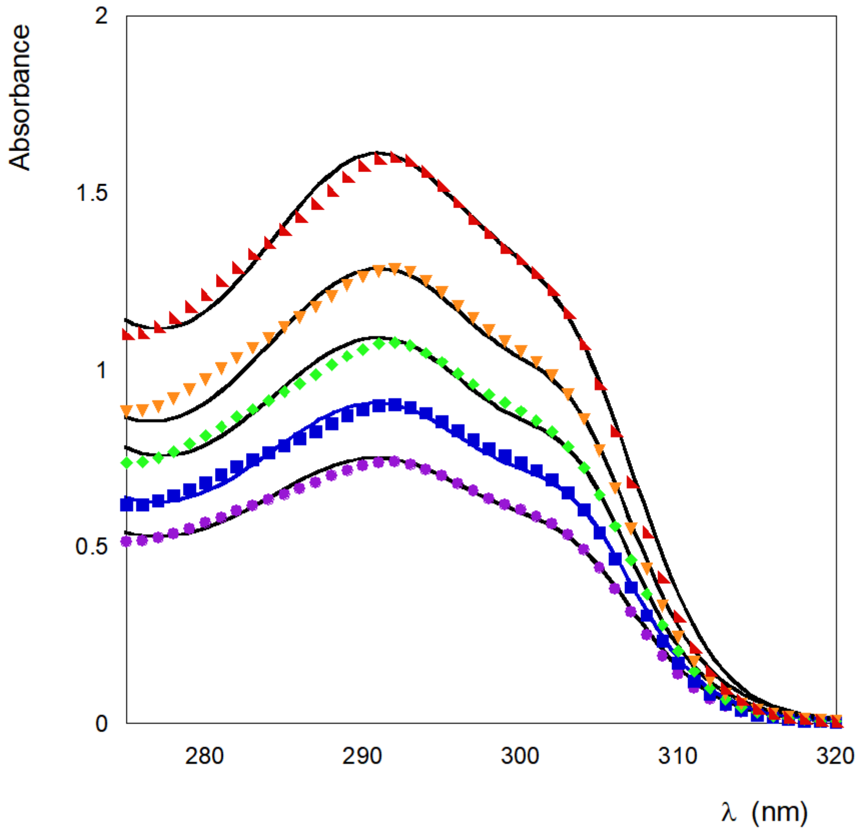

Figure S1. UV-vis absorption spectra of PET in HFIP:$CHCl_3$ 1:1, v:v at different concentration: 0.8 mg/mL (orange line), 1.0 mg/mL (green line), 1.4 mg/mL (blue line), and 1.8 mg/mL (purple line) fitted with the sum of three Gaussian functions centered at 244, 290 and 304 nm.

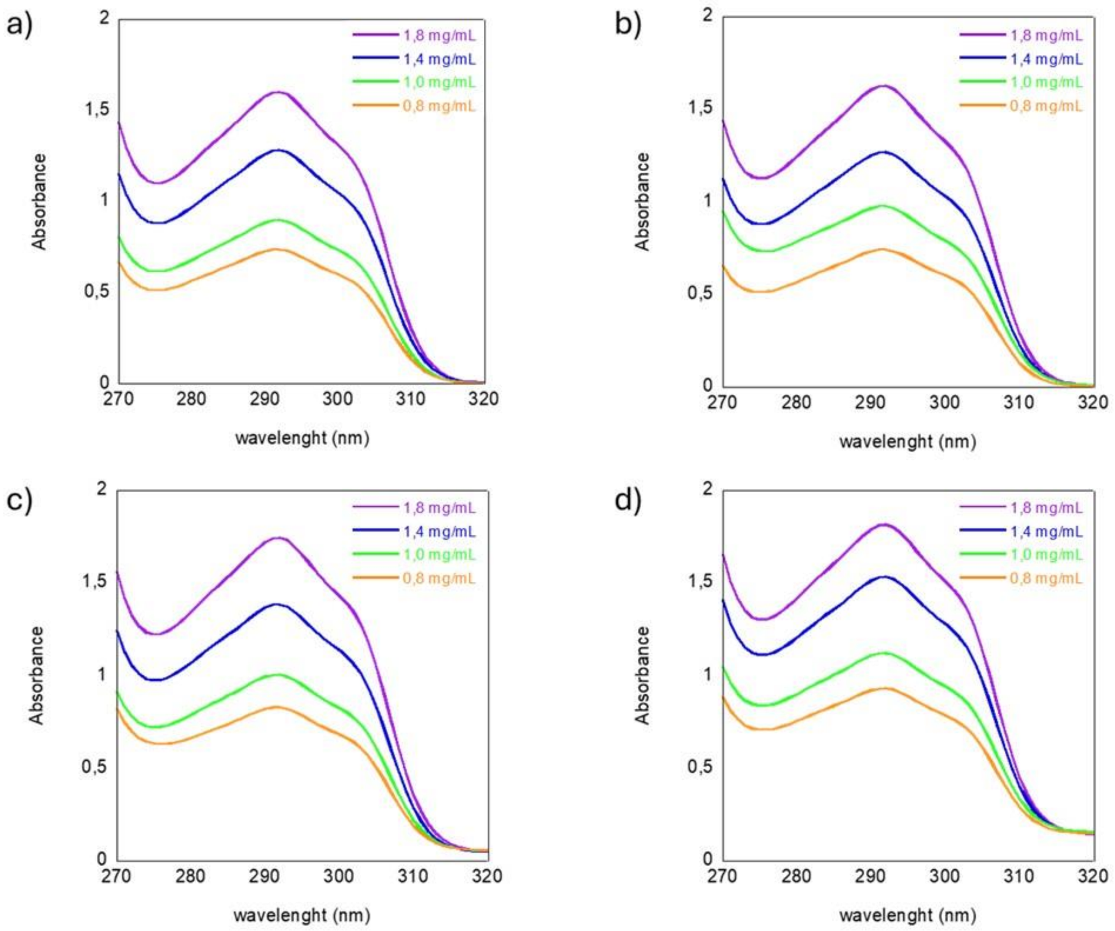

Figure S2. UV-vis absorption spectra of PET in HFIP:$CHCl_3$ 1:1, v:v at different concentration: 0.8 mg/mL (orange line), 1.0 mg/mL (green line), 1.4 mg/mL (blue line), and 1.8 mg/mL (purple line). a) spectra of fresh solutions, b) spectra recorded after 3 days from the preparation, c) spectra recorded after 5 days from the preparation, d) spectra recorded after 8 days from the preparation.

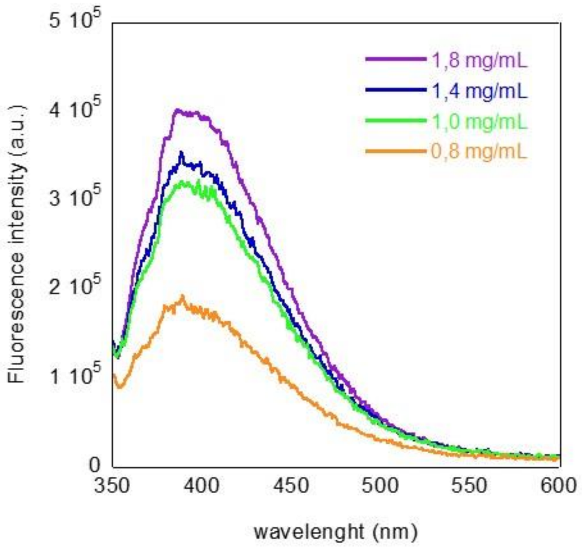

Figure S3. Fluorescence spectra of PET in HFIP:$CHCl_3$ 1:1, v:v at different concentration: 0.8 mg/mL (orange line), 1.0 mg/mL (green line), 1.4 mg/mL (blue line), and 1.8 mg/mL (purple line), λ_{ex}=340 nm. The spectra have been recorded on fresh solutions.

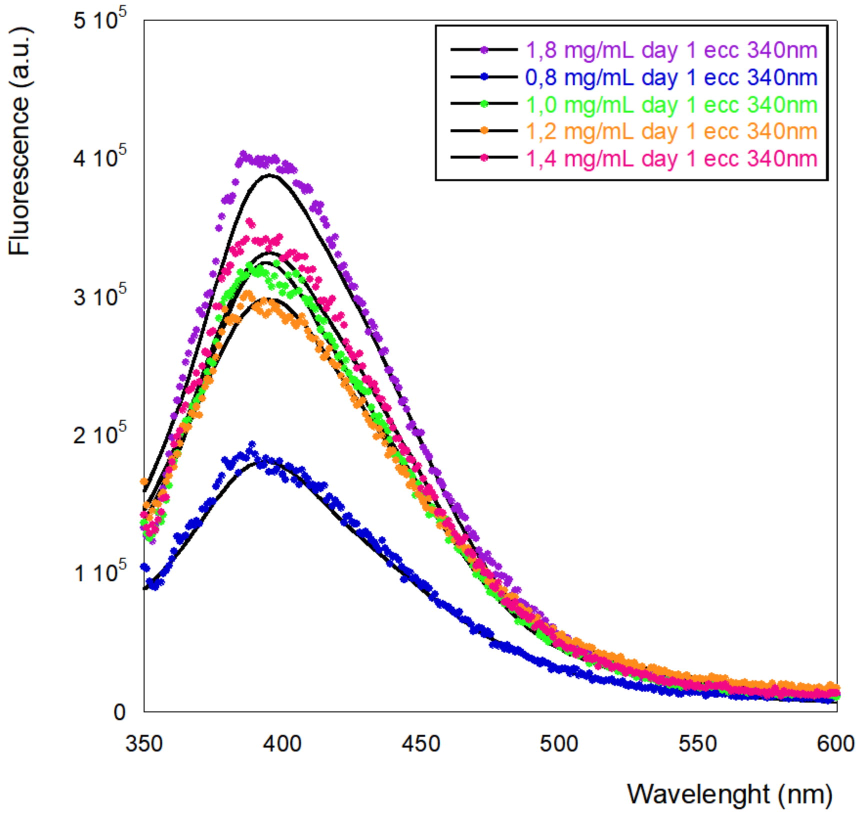

Figure S4. Emission spectra of PET in HFIP:$CHCl_3$ 1:1, v:v at different concentration: 0.8 mg/mL (orange line), 1.0 mg/mL (green line), 1.4 mg/mL (blue line), and 1.8 mg/mL (purple line), λ_{ex}=340 nm recorded the same day of the preparation. The Gaussian fit has been performed using the sum of three Gaussian functions (centered at 367, 390 and 405 nm).

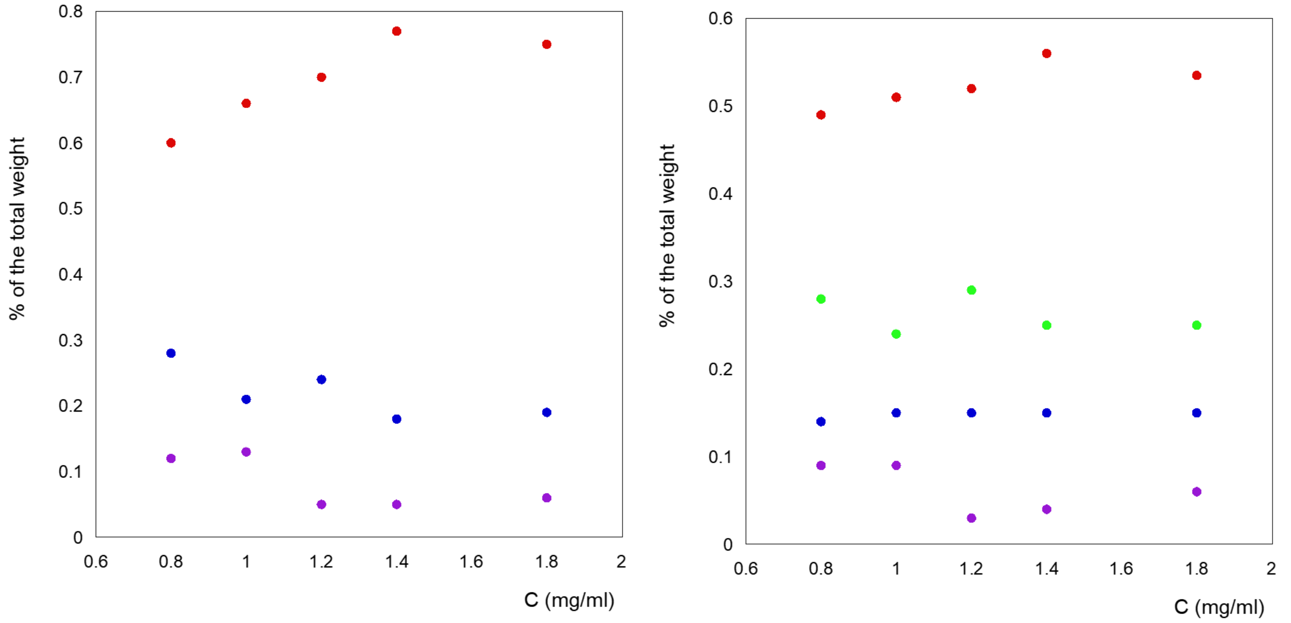

Figure S5. Weight of the single Gaussian function used for the deconvolution fit of emission spectra. Left: weight in function of the concentration at the day 1. Right: weight in function of the concentration at the day 8. Violet is the weight of the Gaussian centered at 367 nm, blue is the weight of the Gaussian centered at 390 nm, red is the weight of the Gaussian centered at 405 nm, and green is the weight of the Gaussian centered at 360 nm.

In particular, we observed that the emission spectra recorded at the day 1, may be well fitted with three Gaussian functions, centered at 367, 390, 405 nm. Furthermore, we observed that the weight of the peak centered at 367 and 390, at the day 1, decrease by increasing the concentration, while the weight of the 405 nm band increases. The day 8, instead, the fit works with a fourth Gaussian function, which is located at 460 nm. 460 nm is the region of the monomer emission: we find the formation of small species in DLS measurements.

Dynamic Light Scattering measurements

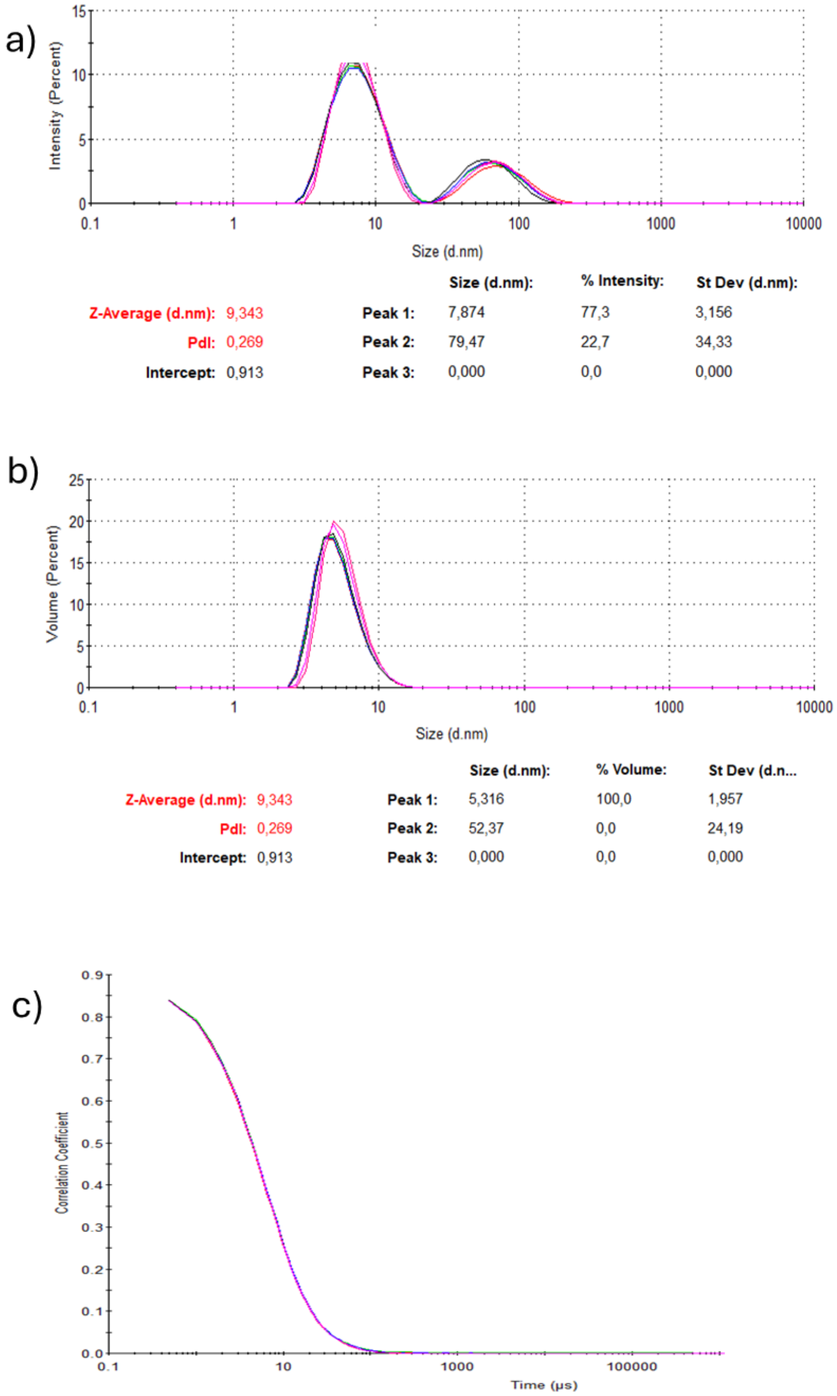

Figure S6.1 DLS measurements performed on 0.8 mg/mL fresh solutions of PET in HFIP:$CHCl_3$ 1:1, v:v. a) Size distribution by Intensity b) Size distribution by Volume c) Raw Correlation data.

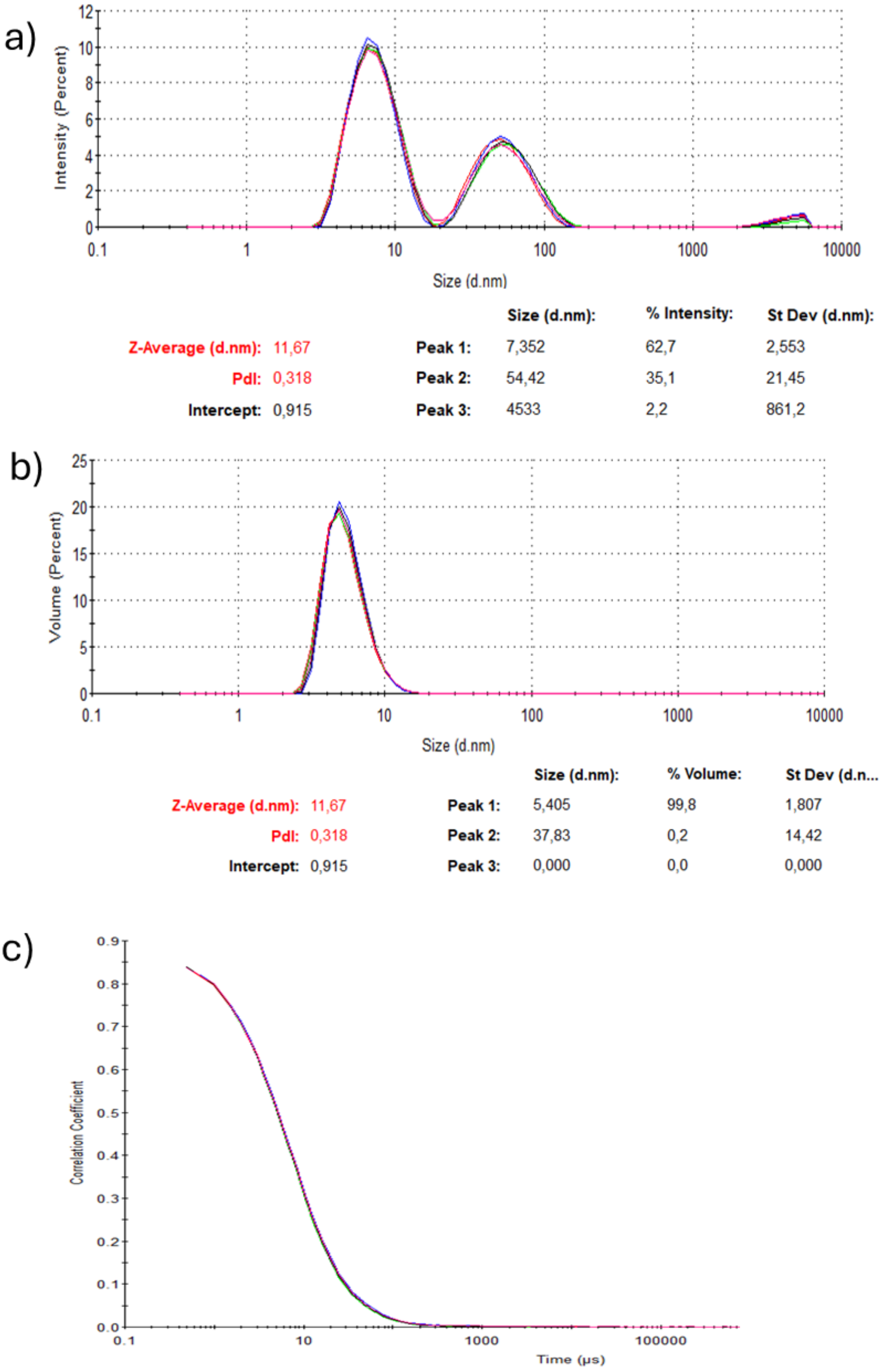

Figure S6.2 DLS measurements performed on 1.0 mg/mL fresh solutions of PET in HFIP:$CHCl_3$ 1:1, v:v. a) Size distribution by Intensity b) Size distribution by Volume c) Raw Correlation data.

a)

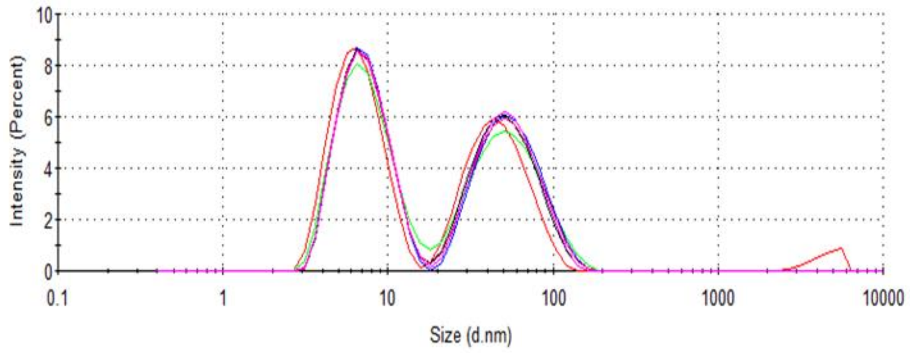

			Size (d.nm):	% Intensity:	St Dev (d.nm):
Z-Average (d.nm):	13,08	Peak 1:	6,726	53,6	2,270
PdI:	0,350	Peak 2:	48,71	43,6	19,80
Intercept:	0,915	Peak 3:	4580	2,8	840,2

b)

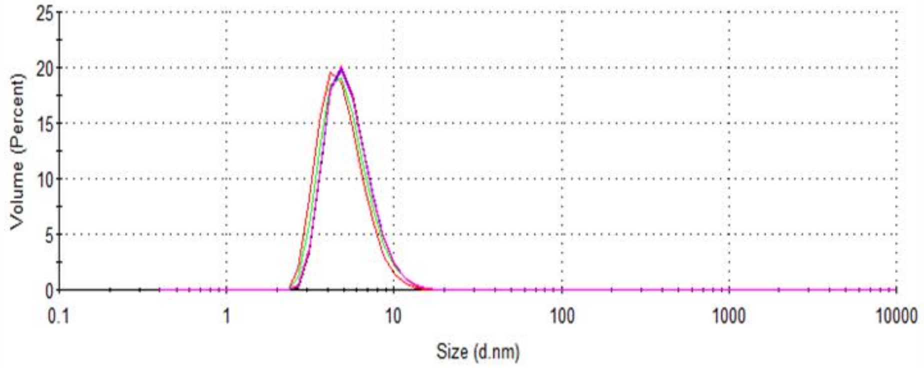

			Size (d.nm):	% Volume:	St Dev (d.n...
Z-Average (d.nm):	13,08	Peak 1:	5,027	99,7	1,644
PdI:	0,350	Peak 2:	32,86	0,3	12,83
Intercept:	0,915	Peak 3:	0,000	0,0	0,000

c)

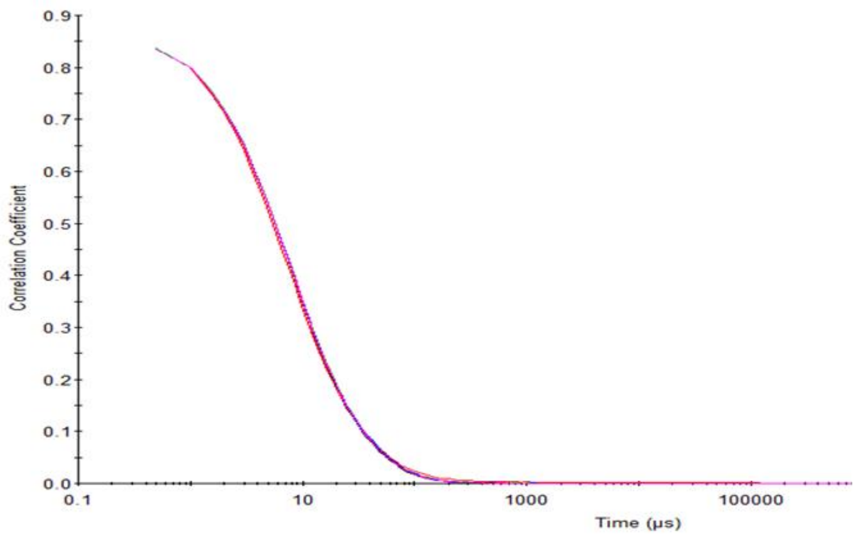

Figure S6.3 DLS measurements performed on 1.4 mg/mL fresh solutions of PET in HFIP:$CHCl_3$ 1:1, v:v. a) Size distribution by Intensity b) Size distribution by Volume c) Raw Correlation data.

a)

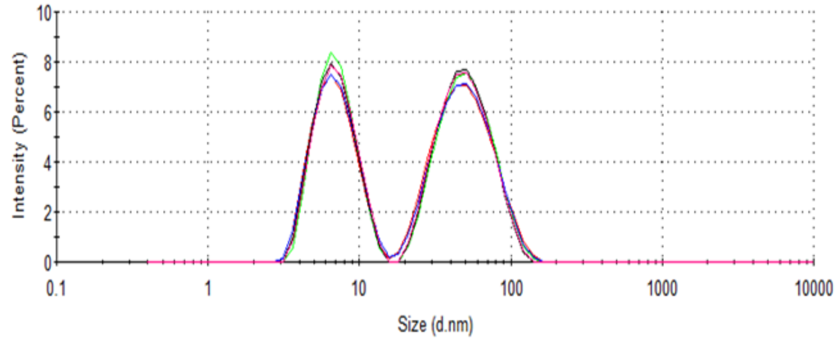

			Size (d.nm):	% Intensity:	St Dev (d.nm):
Z-Average (d.nm):	15,58	Peak 1:	52,66	56,5	23,03
PdI:	0,375	Peak 2:	6,994	43,5	2,258
Intercept:	0,914	Peak 3:	0,000	0,0	0,000

b)

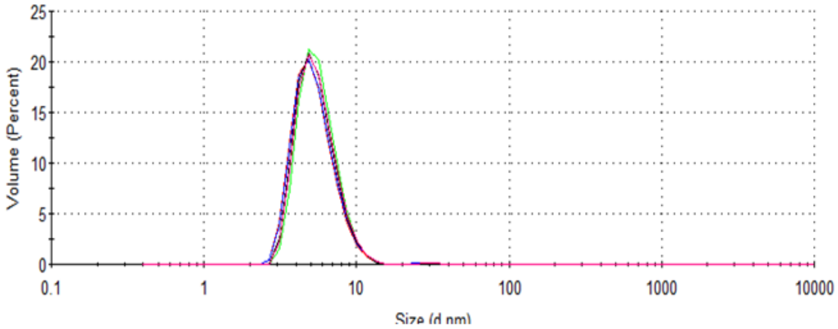

			Size (d.nm):	% Volume:	St Dev (d.n...
Z-Average (d.nm):	15,58	Peak 1:	33,64	0,5	14,08
PdI:	0,375	Peak 2:	5,365	99,5	1,690
Intercept:	0,914	Peak 3:	0,000	0,0	0,000

c)

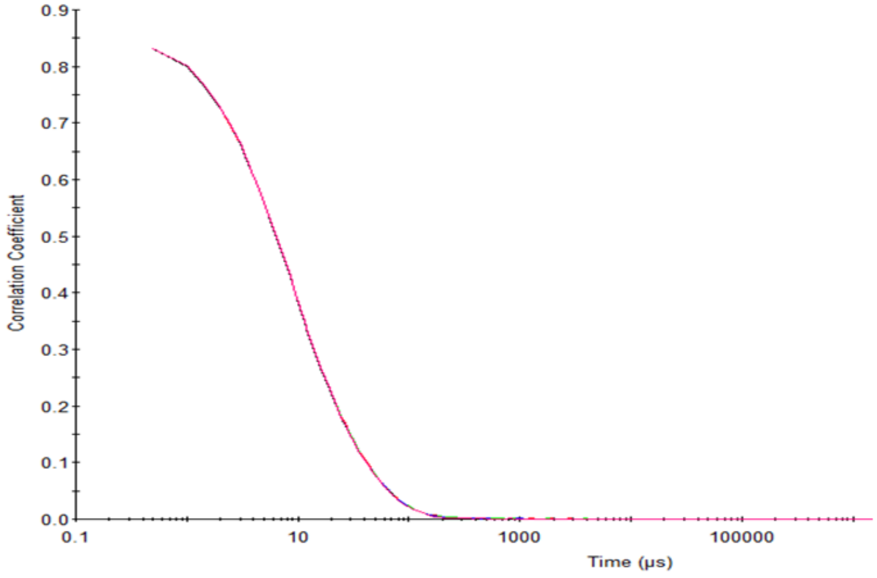

Figure S6.4 DLS measurements performed on 1.8 mg/mL fresh solutions of PET in HFIP:$CHCl_3$ 1:1, v:v. a) Size distribution by Intensity b) Size distribution by Volume c) Raw Correlation data.

a)

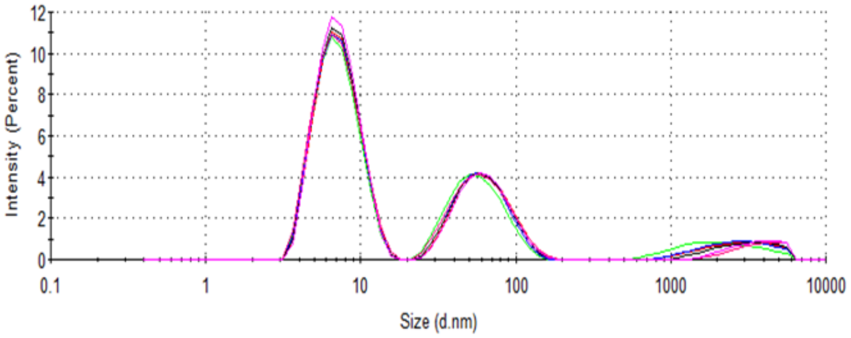

			Size (d.nm):	% Intensity:	St Dev (d.nm):
Z-Average (d.nm):	11,90	Peak 1:	7,305	65,0	2,210
PdI:	0,330	Peak 2:	65,08	28,9	24,58
Intercept:	0,918	Peak 3:	3667	6,1	1183

b)

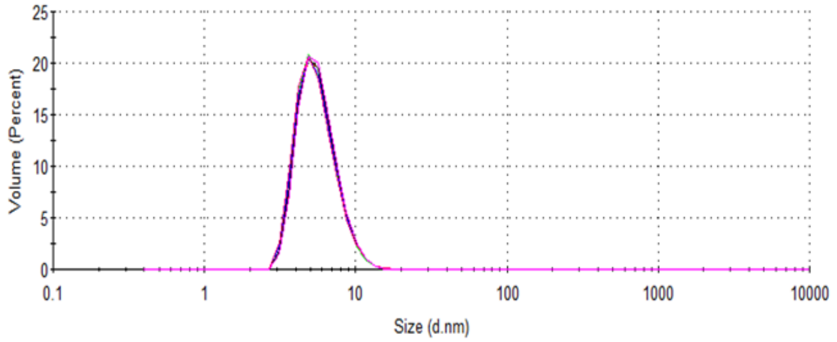

			Size (d.nm):	% Volume:	St Dev (d.n...
Z-Average (d.nm):	11,90	Peak 1:	5,758	99,9	1,720
PdI:	0,330	Peak 2:	46,48	0,1	17,81
Intercept:	0,918	Peak 3:	3843	0,0	1275

c)

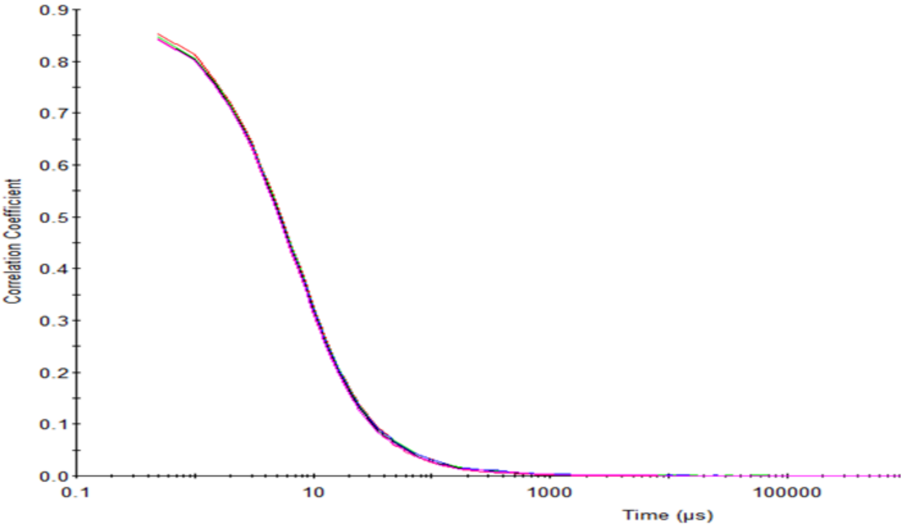

Figure S6.5 DLS measurements performed on 0.8 mg/mL solutions of PET in HFIP:$CHCl_3$ 1:1, v:v. after 3 days from the preparation. a) Size distribution by Intensity b) Size distribution by Volume c) Raw Correlation data.

a)

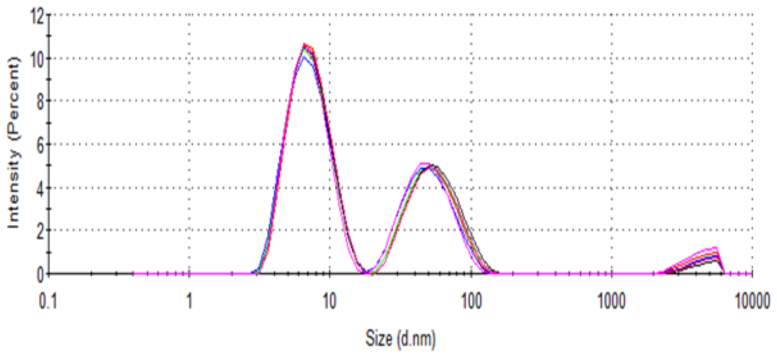

			Size (d.nm):	% Intensity:	St Dev (d.nm):
Z-Average (d.nm):	12,41	Peak 1:	7,162	60,5	2,243
PdI:	0,338	Peak 2:	51,42	34,5	18,70
Intercept:	0,914	Peak 3:	4314	5,0	960,4

b)

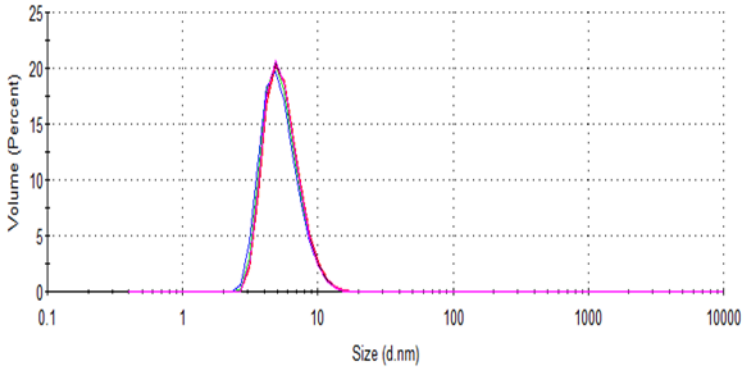

			Size (d.nm):	% Volume:	St Dev (d.n...
Z-Average (d.nm):	12,41	Peak 1:	5,560	99,8	1,701
PdI:	0,338	Peak 2:	36,63	0,2	13,59
Intercept:	0,914	Peak 3:	4455	0,0	1027

c)

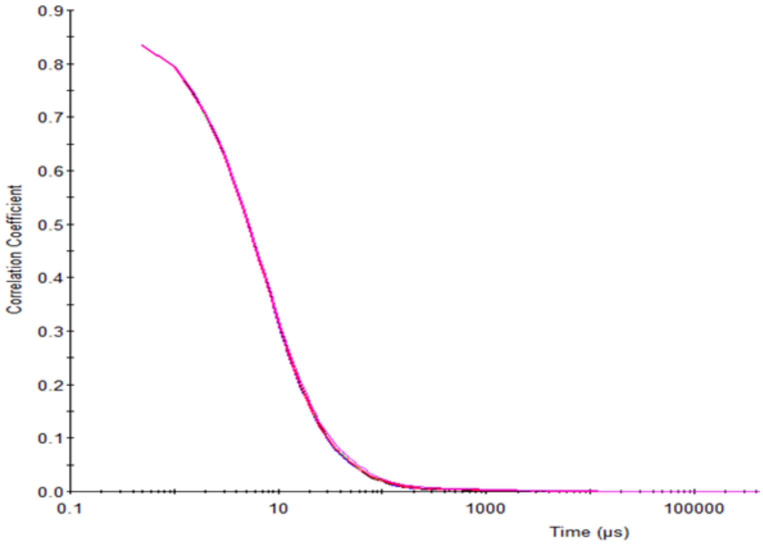

Figure S6.6 DLS measurements performed on 1.0 mg/mL solutions of PET in HFIP:$CHCl_3$ 1:1, v:v. after 3 days from the preparation. a) Size distribution by Intensity b) Size distribution by Volume c) Raw Correlation data.

a)

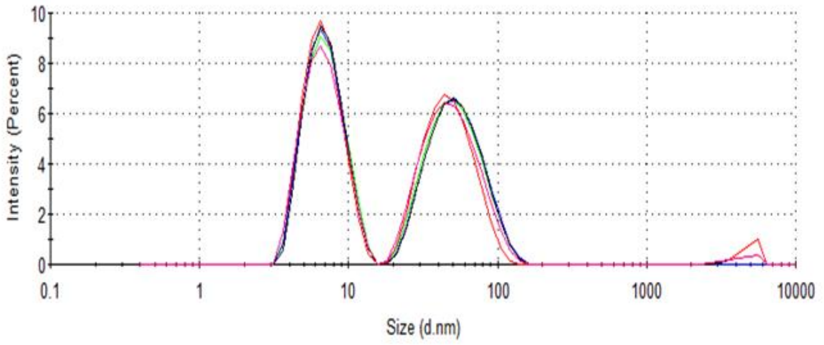

			Size (d.nm):	% Intensity:	St Dev (d.nm):
Z-Average (d.nm):	14,19	Peak 1:	51,82	49,4	21,93
PdI:	0,359	Peak 2:	6,975	49,2	2,152
Intercept:	0,946	Peak 3:	4435	1,3	906,6

b)

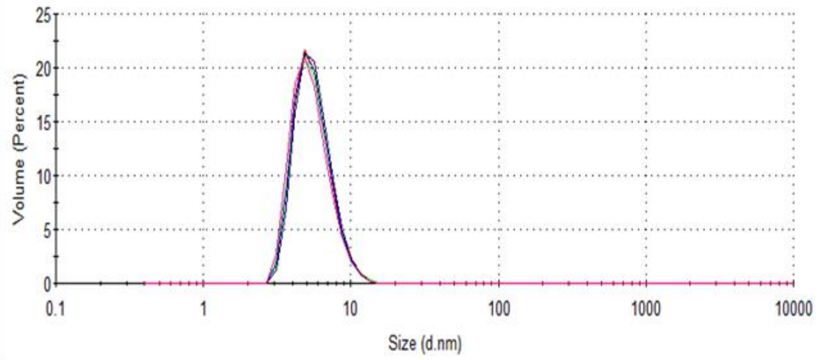

			Size (d.nm):	% Volume:	St Dev (d.n...
Z-Average (d.nm):	14,19	Peak 1:	5,456	99,6	1,654
PdI:	0,359	Peak 2:	34,24	0,4	13,69
Intercept:	0,946	Peak 3:	0,000	0,0	0,000

c)

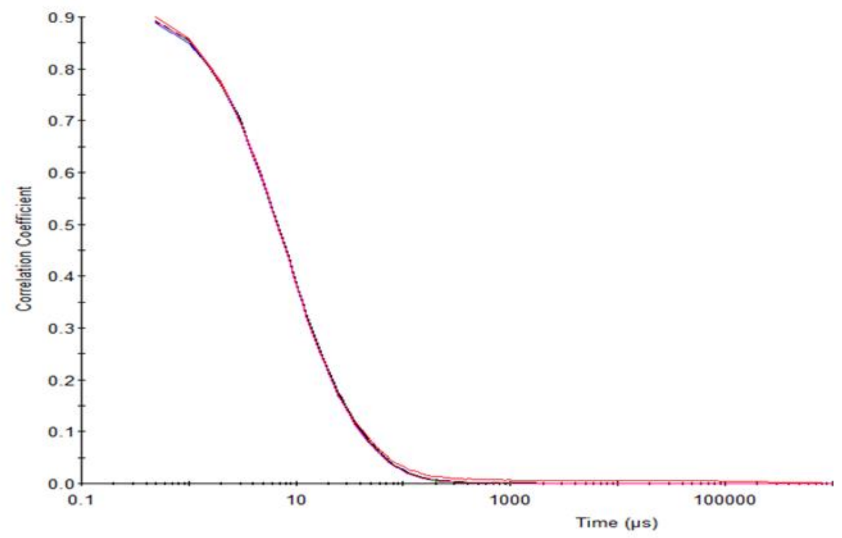

Figure S6.7 DLS measurements performed on 1.4 mg/mL solutions of PET in HFIP:$CHCl_3$ 1:1, v:v. after 3 days from the preparation. a) Size distribution by Intensity b) Size distribution by Volume c) Raw Correlation data.

a)

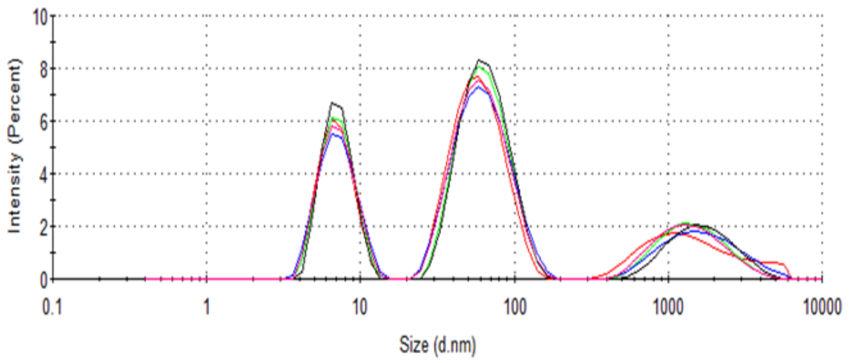

			Size (d.nm):	% Intensity:	St Dev (d.nm):
Z-Average (d.nm):	28,55	Peak 1:	64,56	53,3	25,11
PdI:	0,622	Peak 2:	7,219	28,0	1,860
Intercept:	0,809	Peak 3:	1512	18,7	759,4

b)

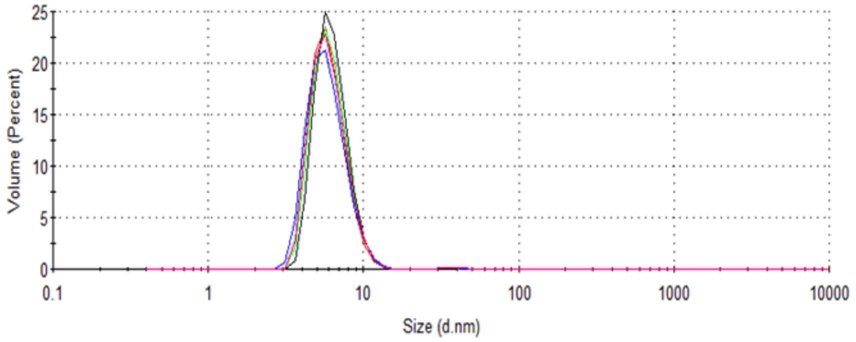

			Size (d.nm):	% Volume:	St Dev (d.n...
Z-Average (d.nm):	28,55	Peak 1:	45,18	0,5	17,78
PdI:	0,622	Peak 2:	6,049	99,4	1,601
Intercept:	0,809	Peak 3:	1523	0,2	730,3

c)

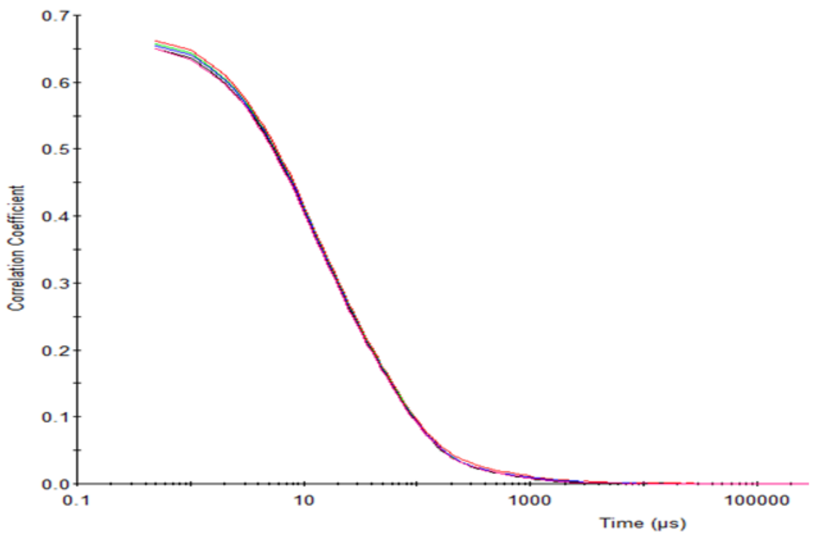

Figure S6.8 DLS measurements performed on 1.8 mg/mL solutions of PET in HFIP:$CHCl_3$ 1:1, v:v. after 3 days from the preparation. a) Size distribution by Intensity b) Size distribution by Volume c) Raw Correlation data.

a)

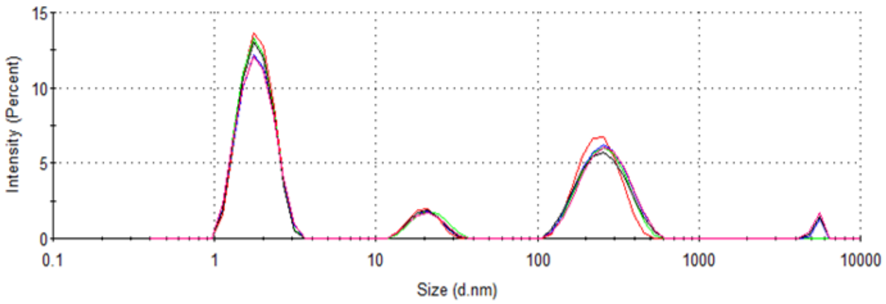

			Size (d.nm):	% Intensity:	St Dev (d.nm):
Z-Average (d.nm):	76,11	Peak 1:	1,851	54,9	0,4490
PdI:	0,370	Peak 2:	274,6	36,1	87,27
Intercept:	0,859	Peak 3:	20,96	6,9	4,529

b)

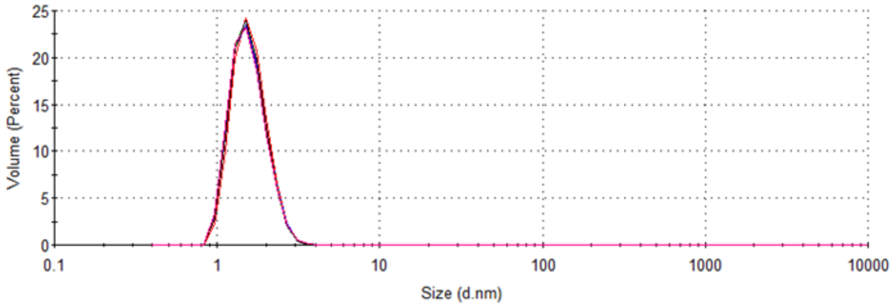

			Size (d.nm):	% Volume:	St Dev (d.n...
Z-Average (d.nm):	76,11	Peak 1:	1,578	100,0	0,4017
PdI:	0,370	Peak 2:	0,000	0,0	0,000
Intercept:	0,859	Peak 3:	0,000	0,0	0,000

c)

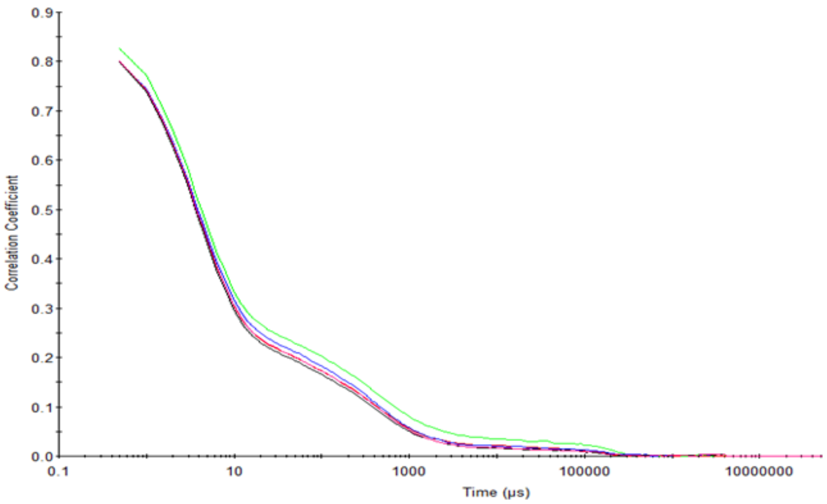

Figure S6.9 DLS measurements performed on 0.8 mg/mL solutions of PET in HFIP:$CHCl_3$ 1:1, v:v. after 5 days from the preparation. a) Size distribution by Intensity b) Size distribution by Volume c) Raw Correlation data.

a)

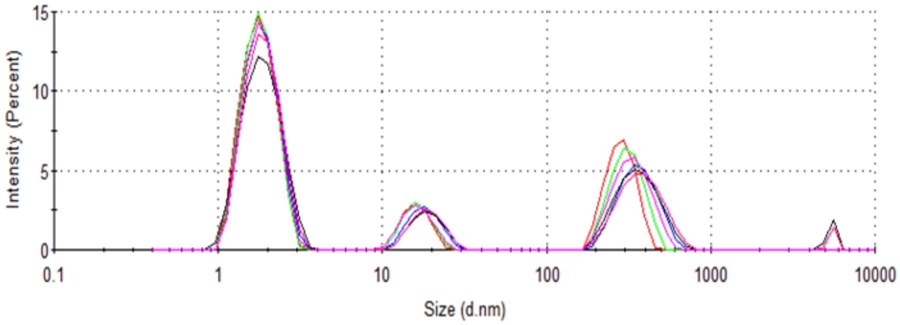

			Size (d.nm):	% Intensity:	St Dev (d.nm):
Z-Average (d.nm):	118,7	Peak 1:	1,834	61,8	0,4216
PdI:	0,298	Peak 2:	335,6	27,5	83,56
Intercept:	0,859	Peak 3:	16,40	10,7	3,336

b)

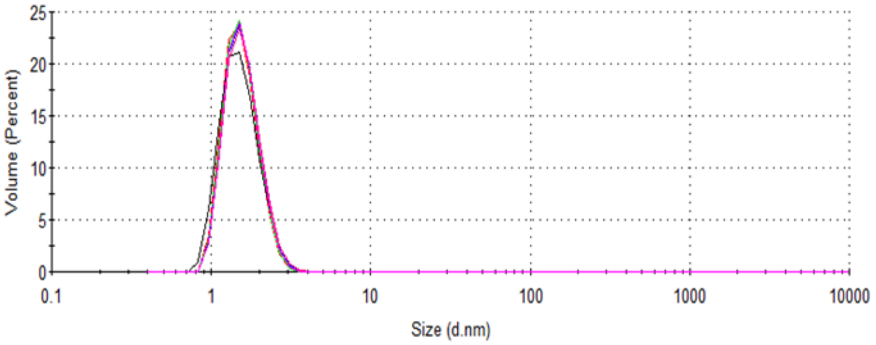

			Size (d.nm):	% Volume:	St Dev (d.n...
Z-Average (d.nm):	118,7	Peak 1:	1,586	100,0	0,3899
PdI:	0,298	Peak 2:	14,62	0,0	3,308
Intercept:	0,859	Peak 3:	0,000	0,0	0,000

c)

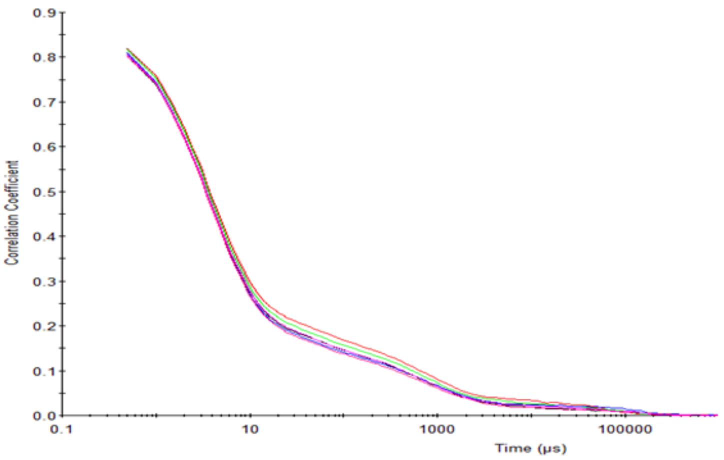

Figure S6.10 DLS measurements performed on 1.0 mg/mL solutions of PET in HFIP:$CHCl_3$ 1:1, v:v. after 5 days from the preparation. a) Size distribution by Intensity b) Size distribution by Volume c) Raw Correlation data.

a)

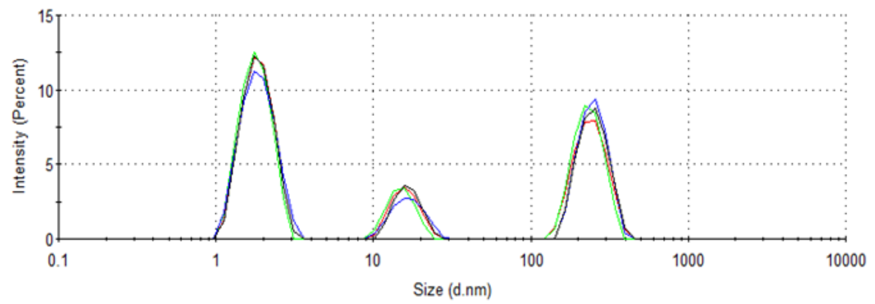

			Size (d.nm):	% Intensity:	St Dev (d.nm):
Z-Average (d.nm):	201,0	Peak 1:	1,867	51,7	0,4164
PdI:	0,311	Peak 2:	252,0	35,5	53,71
Intercept:	0,908	Peak 3:	16,58	12,7	3,094

b)

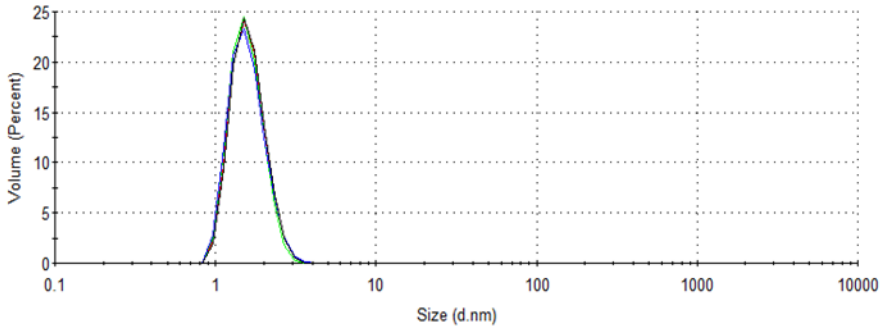

			Size (d.nm):	% Volume:	St Dev (d.n...
Z-Average (d.nm):	201,0	Peak 1:	1,626	100,0	0,3937
PdI:	0,311	Peak 2:	15,10	0,0	3,150
Intercept:	0,908	Peak 3:	0,000	0,0	0,000

c)

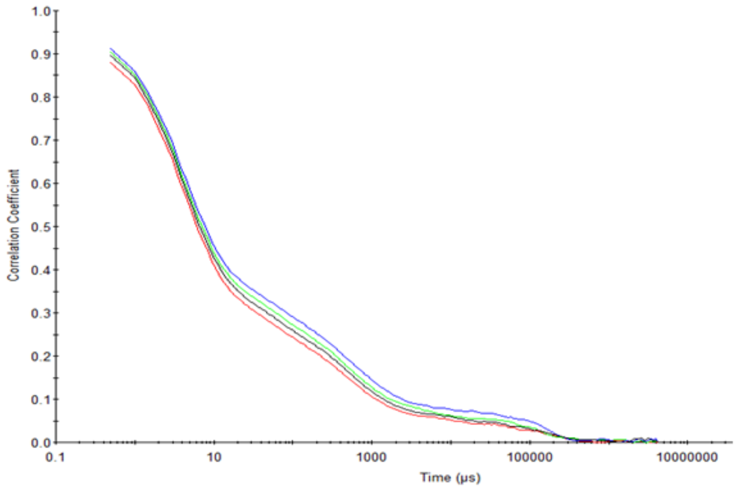

Figure S6.11 DLS measurements performed on 1.4 mg/mL solutions of PET in HFIP:$CHCl_3$ 1:1, v:v. after 5 days from the preparation. a) Size distribution by Intensity b) Size distribution by Volume c) Raw Correlation data.

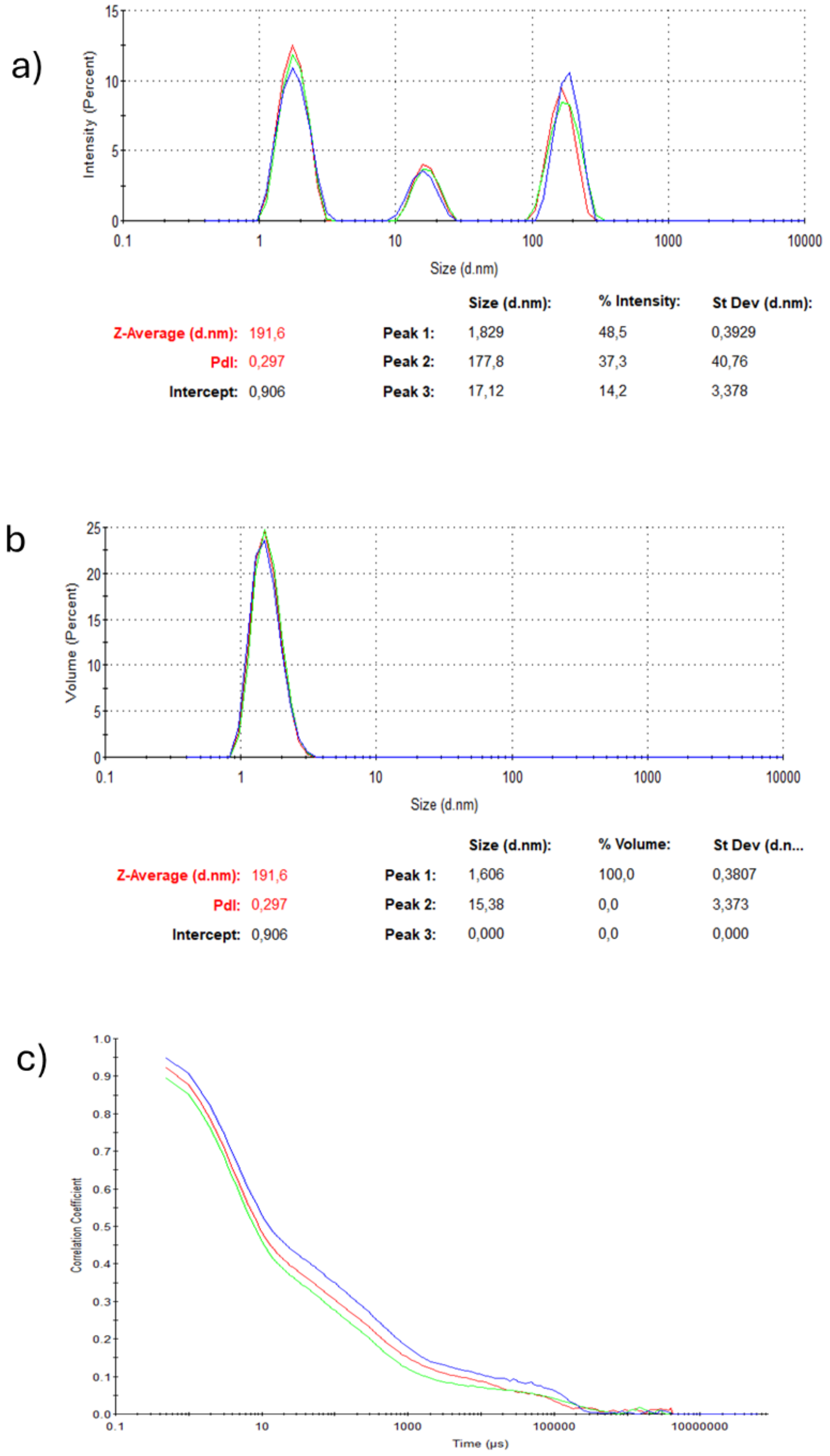

Figure S6.12 DLS measurements performed on 1.8 mg/mL solutions of PET in HFIP:$CHCl_3$ 1:1, v:v. after 5 days from the preparation. a) Size distribution by Intensity b) Size distribution by Volume c) Raw Correlation data.

a)

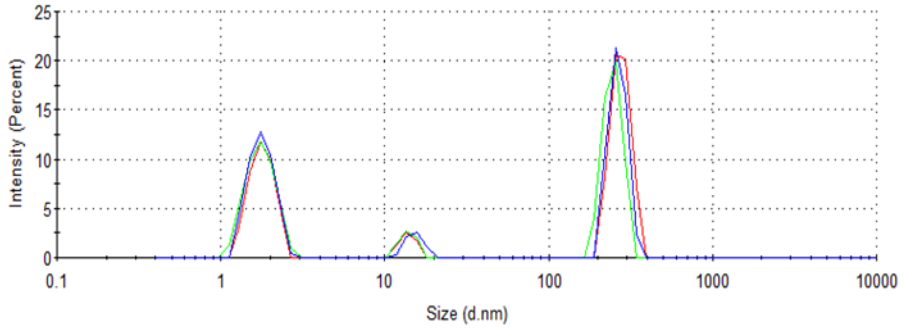

			Size (d.nm):	% Intensity:	St Dev (d.nm):
Z-Average (d.nm):	744,2	Peak 1:	264,8	50,8	32,23
PdI:	0,729	Peak 2:	1,783	43,1	0,3153
Intercept:	0,989	Peak 3:	15,18	6,1	1,809

b)

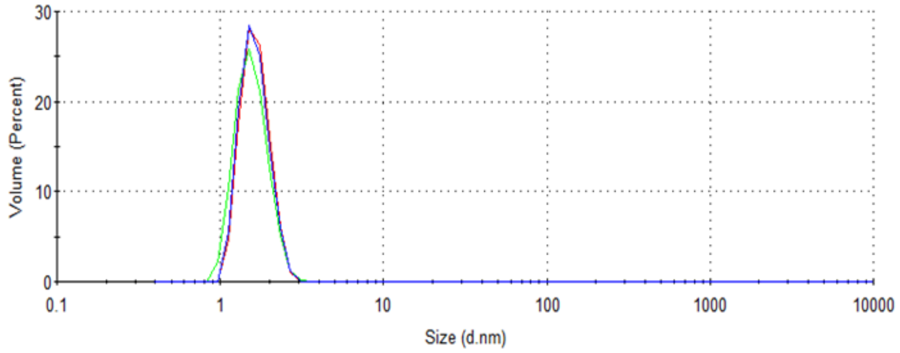

			Size (d.nm):	% Volume:	St Dev (d.n...
Z-Average (d.nm):	744,2	Peak 1:	1,638	100,0	0,3294
PdI:	0,729	Peak 2:	14,62	0,0	2,325
Intercept:	0,989	Peak 3:	0,000	0,0	0,000

c)

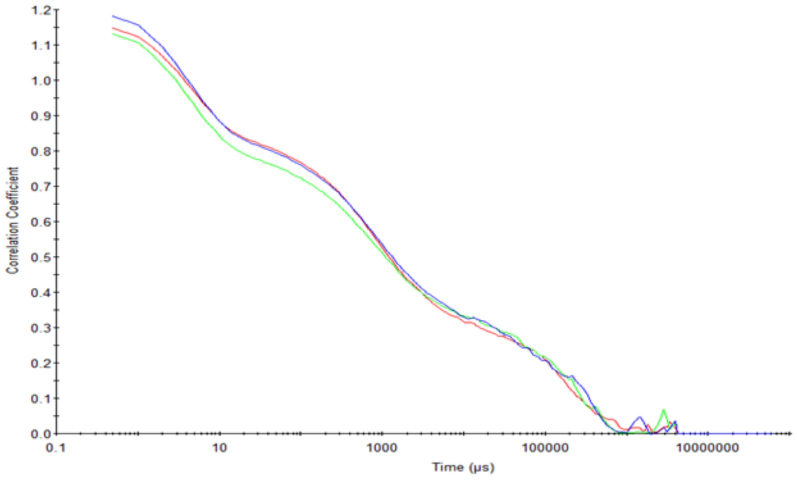

Figure S6.13 DLS measurements performed on 0.8 mg/mL solutions of PET in HFIP:$CHCl_3$ 1:1, v:v. after 8 days from the preparation. a) Size distribution by Intensity b) Size distribution by Volume c) Raw Correlation data.

a)

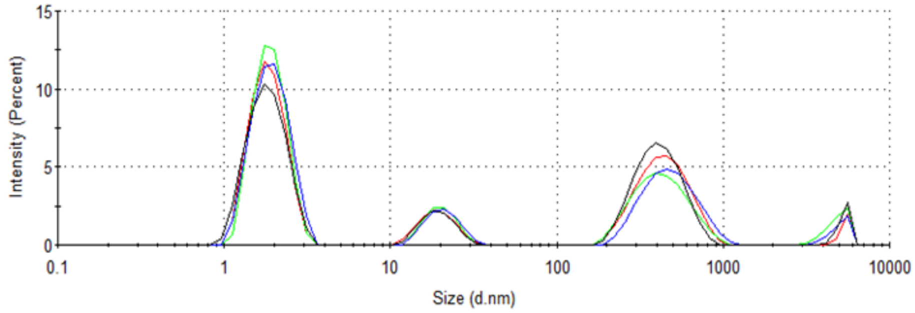

			Size (d.nm):	% Intensity:	St Dev (d.nm):
Z-Average (d.nm):	92,97	Peak 1:	1,845	49,3	0,4723
PdI:	0,529	Peak 2:	420,5	37,8	130,4
Intercept:	0,882	Peak 3:	19,45	9,1	4,298

b)

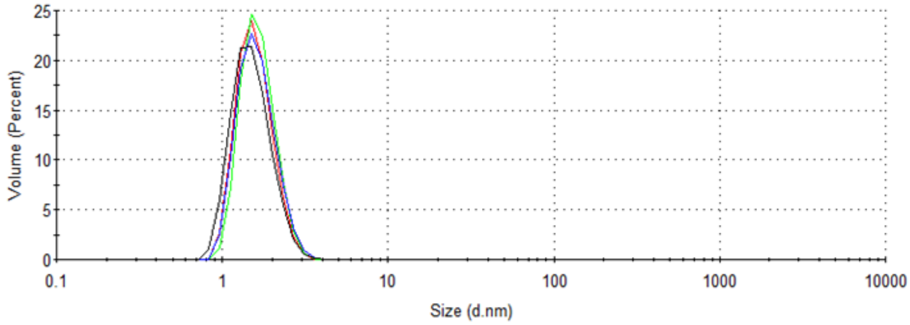

			Size (d.nm):	% Volume:	St Dev (d.n...
Z-Average (d.nm):	119,7	Peak 1:	1,600	100,0	0,3985
PdI:	0,445	Peak 2:	16,78	0,0	4,185
Intercept:	0,877	Peak 3:	0,000	0,0	0,000

c)

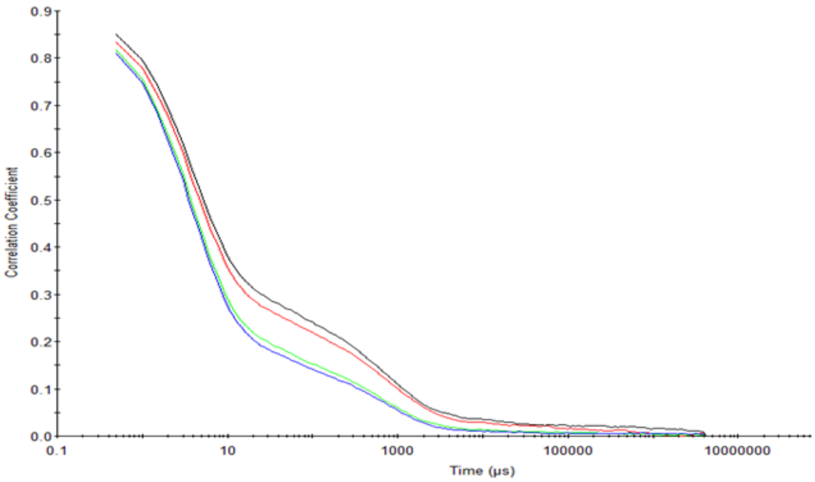

Figure S6.14 DLS measurements performed on 1.0 mg/mL solutions of PET in HFIP:$CHCl_3$ 1:1, v:v. after 8 days from the preparation. a) Size distribution by Intensity b) Size distribution by Volume c) Raw Correlation data.

a)

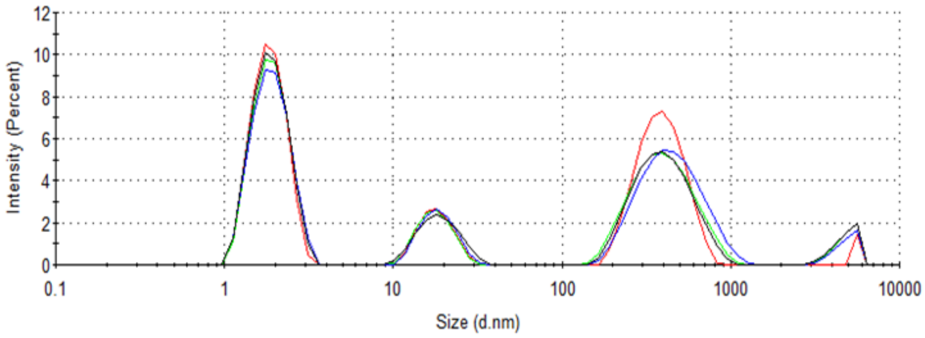

			Size (d.nm):	% Intensity:	St Dev (d.nm):
Z-Average (d.nm):	31,59	Peak 1:	1,898	45,3	0,4527
PdI:	0,550	Peak 2:	414,3	37,9	157,9
Intercept:	0,873	Peak 3:	19,00	11,9	5,119

b)

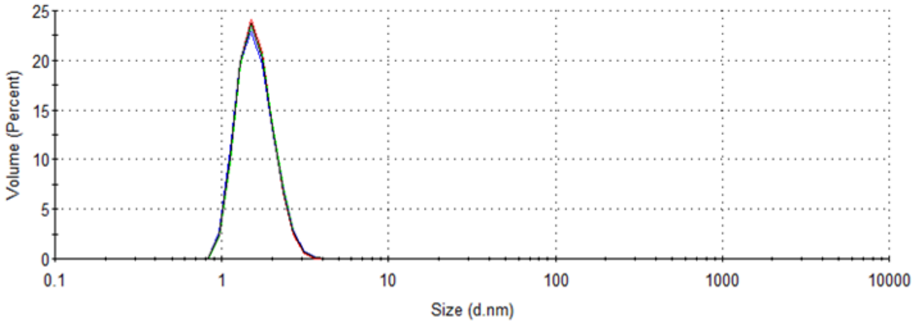

			Size (d.nm):	% Volume:	St Dev (d.n...
Z-Average (d.nm):	135,3	Peak 1:	1,621	100,0	0,3970
PdI:	0,488	Peak 2:	15,90	0,0	3,750
Intercept:	0,895	Peak 3:	0,000	0,0	0,000

c)

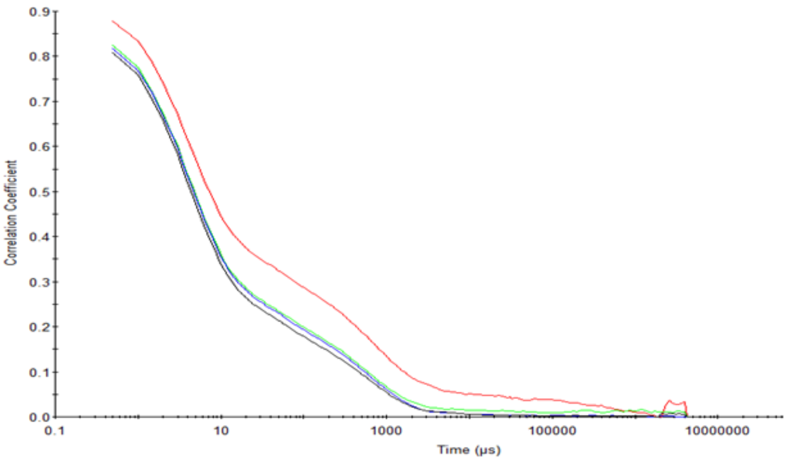

Figure S6.15 DLS measurements performed on 1.4 mg/mL solutions of PET in HFIP:$CHCl_3$ 1:1, v:v. after 8 days from the preparation. a) Size distribution by Intensity b) Size distribution by Volume c) Raw Correlation data.

a)

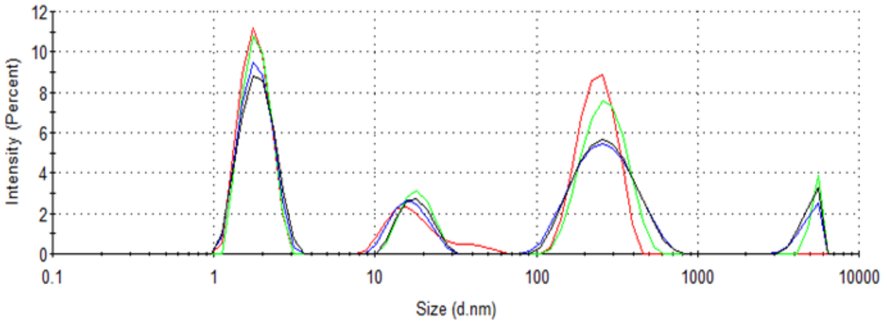

			Size (d.nm):	% Intensity:	St Dev (d.nm):
Z-Average (d.nm):	64,17	Peak 1:	283,2	42,1	115,2
PdI:	0,552	Peak 2:	1,896	39,2	0,4439
Intercept:	0,883	Peak 3:	18,06	11,6	3,997

b)

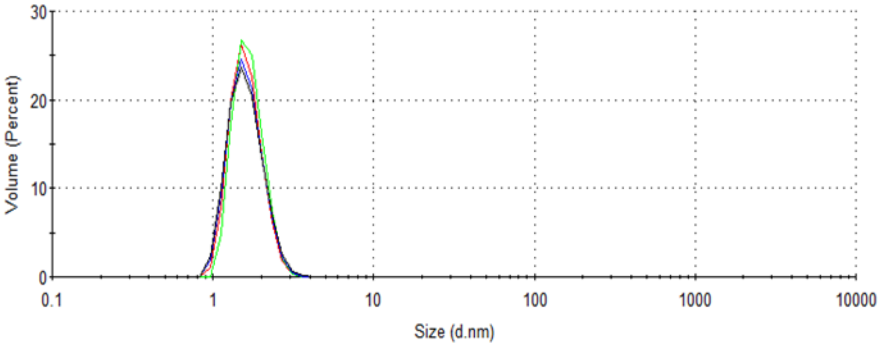

			Size (d.nm):	% Volume:	St Dev (d.n...
Z-Average (d.nm):	223,7	Peak 1:	1,625	100,0	0,3640
PdI:	0,337	Peak 2:	13,61	0,0	4,396
Intercept:	0,905	Peak 3:	0,000	0,0	0,000

c)

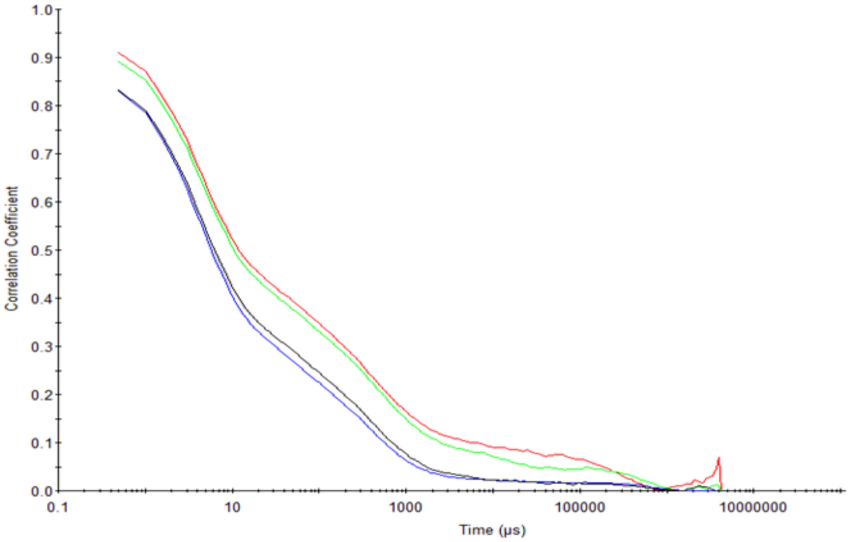

Figure S6.16 DLS measurements performed on 1.8 mg/mL solutions of PET in HFIP:$CHCl_3$ 1:1, v:v. after 8 days from the preparation. a) Size distribution by Intensity b) Size distribution by Volume c) Raw Correlation data.

C = 0.8 mg/mL			
**Day**	**Size**	**DS**	**I%**
**1**	7,874	3,156	77,3
	79,47	34,33	22,7
**3**	7,305	2,210	65
	65,08	24,58	28,9
**5**	1,851	0,449	54,9
	20,96	4,529	6,9
	274,6	36,1	87,27
**8**	1,783	0,315	43,1
	15,18	1,81	6,1
	264,8	32,23	50,8

C = 1.0 mg/mL			
**Day**	**Size**	**DS**	**I%**
**1**	7,352	2,553	62,7
	54,48	21,45	35,1
**3**	7,162	2,243	60,5
	51,42	18,7	34,5
**5**	1,834	0,422	61,8
	16,4	3,336	10,7
	335,6	83,56	27,5
**8**	1,845	0,47	49,3
	19,45	4,30	9,1
	420,5	130,4	37,8

C = 1.4 mg/mL			
**Day**	**Size**	**DS**	**I%**
**1**	6,726	2,27	53,6
	48,71	19,8	43,6
**3**	6,975	2,152	49,2
	51,82	21,93	49,4
**5**	1,867	0,416	51,7
	16,58	3,09	12,7
	252	53,71	35,5
**8**	1,90	0,45	45,3
	19,0	5,12	11,9
	414,3	157,9	37,9

C = 1.8 mg/mL			
**Day**	**Size**	**DS**	**I%**
**1**	6,994	2,258	43,5
	52,66	23,03	56,5
**3**	7,219	1,86	28
	64,56	25,11	53,3
**5**	1,829	0,393	48,5
	17,12	3,378	14,2
	177,8	40,76	37,3
**8**	1,90	0,44	39,2
	18,06	4,0	11,6
	283,2	115,2	42,1

Table S1. DLS data (size, standard deviation and distribution by Intensity), grouped by solution concentration. The direct comparison highlights the evolution of the particles size over time.

DLS measurements

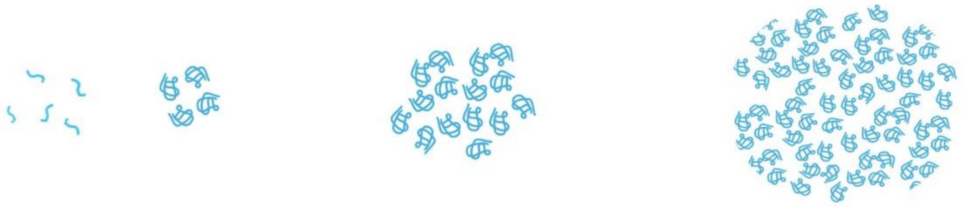

		Size (nm)			
C = 0.8 mg/mL	Day 1		7.9	79.5	
	Day 3		7.3	65.1	
	Day 5	1.9		21.0	274.6
	Day 8	1.8		15.2	264.8
C = 1.0 mg/mL	Day 1		7.4	54.5	
	Day 3		7.2	51.4	
	Day 5	1.8		16.4	335.6
	Day 8	1.8		19.5	420.5
C = 1.4 mg/mL	Day 1		6.7	48.7	
	Day 3		7.0	51.8	
	Day 5	1.9		16.6	252.0
	Day 8	1.9		19.0	414.3
C = 1.8 mg/mL	Day 1		7.0	52.7	
	Day 3		7.2	64.6	
	Day 5	1.8		17.1	177.8
	Day 8	1.9		18.1	283.2

Figure S7. DLS data (size) grouped by solution concentration. The direct comparison highlights the evolution of the particles size over time.

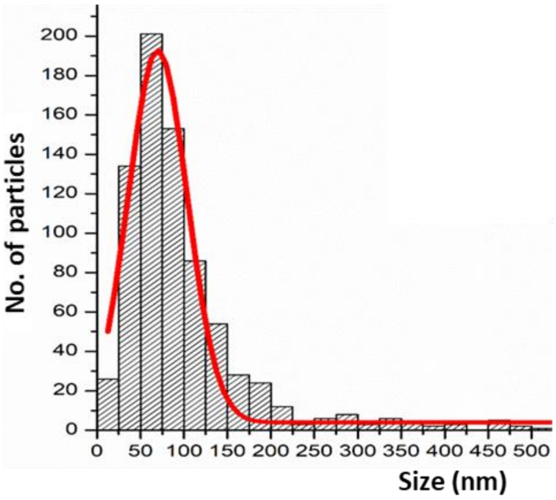

Figure S8. Histogram showing the number of particles and their size, calculated on the acquired SEM images of the PET nanoparticles. Gaussian fit of size distribution is shown in red. The statistical data were collected with *Image J* software.

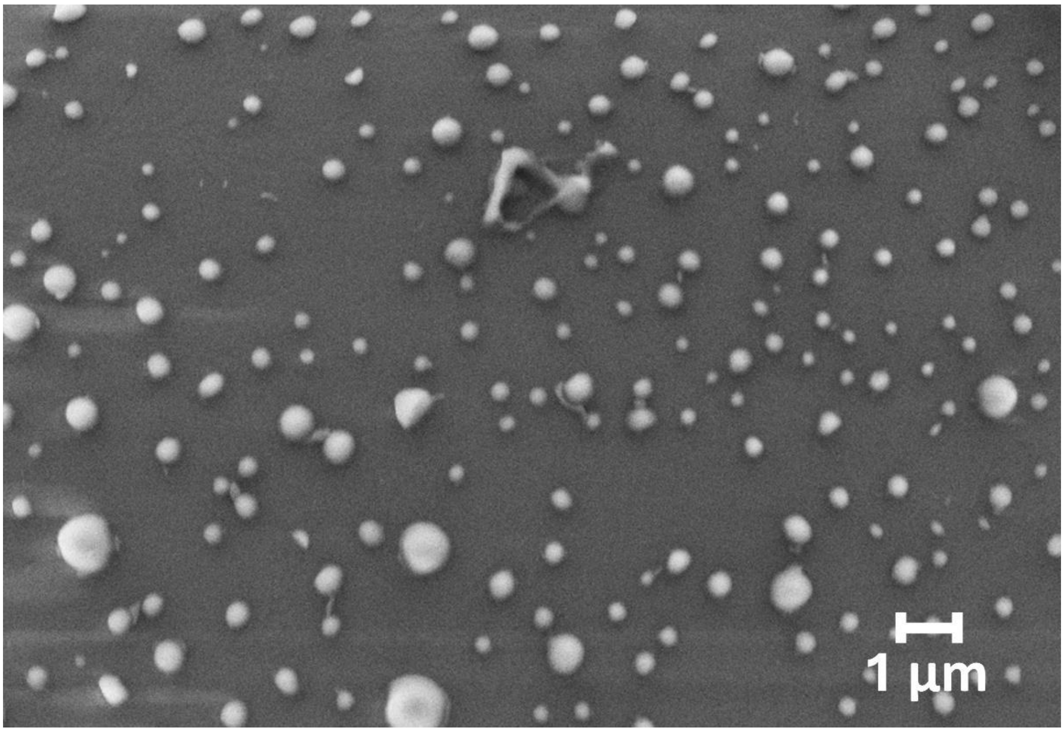

Figure S9. SEM micrograph of PET nanoparticles three months after the preparation.
